# Unlocking Nanocomposites via Cast Film Extrusion: Processing–Structure–Property Relationships of PLA Sheets Containing Silver Nanoparticles

**DOI:** 10.3390/molecules31213808

**Published:** 2026-10-22

**Authors:** Kyriaki Lazaridou, Rafail O. Ioannidis, Panagiotis A. Klonos, Apostolos Kyritsis, Dimitrios N. Bikiaris

**Affiliations:** 1 Laboratory of Polymer Chemistry and Technology, Department of Chemistry, Aristotle University of Thessaloniki, 54124 Thessaloniki, Greece; klazarii@chem.auth.gr (K.L.); rafailio@chem.auth.gr (R.O.I.); pklonos@central.ntua.gr (P.A.K.); 2 Department of Physics, National Technical University of Athens, Zografou Campus, 15780 Athens, Greece; akyrits@central.ntua.gr

**Keywords:** poly(lactic acid) (PLA), silver nanoparticles (Ag NPs), crystallinity, cast film extrusion

## Abstract

A thorough investigation was conducted on poly(lactic acid) (PLA) nanocomposites based on silver (Ag) nanoparticles (NPs) as metallic nanofillers (from 0.5 to 5.0 wt%). The PLA Ag nanocomposite films were developed using the cast film extrusion technique. Gel permeation chromatography and intrinsic viscosity measurements revealed that cast film extrusion induced a significant reduction in the number average molecular weight of PLA, attributed to thermomechanical degradation, whereas the presence of NPs assisted and promoted the stability of the PLA films without further degradation. Proton nuclear magnetic resonance spectroscopy and Fourier-transform infrared spectroscopy confirmed that the incorporation of Ag NPs did not induce any changes to the chemical structure of the polymer matrix. Scanning electron microscopy analysis showed an oriented morphology in neat PLA due to extrusion induced flow, whereas the addition of Ag NPs increased the surface heterogeneity. Differential scanning calorimetry results indicated that Ag NPs acted as nucleating agents, enhancing the degree of crystallinity of PLA. Complex segmental dynamics was revealed by dielectric spectroscopy, with the main segmental relaxation being accelerated and exhibiting an almost monotonic lowering of the cooperativity level with Ag NP loading. Partial densification of the polymer around the NPs and an increase in the free polymer volume away from them was expected. The mechanical performance was evaluated through tensile and flexural tests, where the presence of NPs enhanced the stiffness and strength of the samples, while the ductility was reduced. Annealing experiments also enhanced the mechanical performance of the materials. Finally, water contact angle measurements showed that the surface hydrophobicity was increased. Thus, in this study, PLA nanocomposites based on metal NPs produced via cast film extrusion were developed for advanced industrial applications; such nanocomposites are reported experimentally for the first time.

## 1. Introduction

The growing demand for higher production volumes and improved quality of manufactured polymeric films, together with the introduction of new materials, has led to the need for innovative production approaches. Among the technologies addressing these demands, cast film extrusion, widely used in the film industry, stands out as a continuous, high-speed manufacturing process and holds a prominent position among polymer processing techniques [1,2]. This process produces a wide variety of plastic films and sheets, which find applications in numerous technical fields, including plastic bags, consumer packaging, optical films for liquid crystal displays, flexible electronics, and microporous membranes primarily used in separation processes [3,4]. Although the film extrusion process has been used on a large scale for many years, the production of composite films remains challenging particularly for emerging biodegradable polymers and nanocomposites [5].

Poly(lactic acid) (PLA) is a linear aliphatic polyester which exhibits a number of attractive properties [6]. It is derived from renewable resources, is compostable, has a relatively low cost, and has better thermal properties compared to the conventional polymers [7,8]. Over recent years, PLA has become one of the most widely produced and utilized biobased plastics worldwide [9]. Due to these advantages, PLA is considered a highly promising material for a wide range of applications, including medicine, packaging, agriculture, electronics, and the automotive industry [10,11,12]. However, PLA also presents some drawbacks, such as low melt strength, brittleness, and sensitivity to moisture and gases [13]. Additionally, PLA degrades relatively slowly under natural environmental conditions, as it is primarily compostable rather than readily biodegradable, which may contribute to the formation of microplastics [14].

Moreover, various strategies have been explored over the last few decades to overcome the inherent limitations of PLA, including copolymerization and the incorporation of organic or inorganic fillers. In this regard, Terzopoulou et al. provided a comprehensive overview of a decade of advances in PLA copolymers, highlighting the potential of copolymerization to modify the properties and broaden the application scope of PLA [15]. Alongside copolymerization, the development of PLA nanocomposites represents another promising approach to enhancing the material’s performance. The addition of organic or inorganic fillers provides a potential strategy to address the limited crystallization of PLA and general thermal behavior [16,17,18]. Among these, metal NPs, particularly silver nanoparticles (Ag NPs), have attracted considerable research attention due to their unique physicochemical characteristics. Ag NPs are especially valued for their excellent electrical conductivity and strong antimicrobial activity. These distinctive features make them highly versatile materials with applications in a wide range of fields, including electronics, textiles, sensors, and biomedical technologies [19,20,21,22,23,24,25,26].

Several studies have investigated the incorporation of Ag NPs into PLA matrices, mainly focusing on the enhancement in antimicrobial activity and functional performance. PLA/Ag nanocomposites have been extensively reported for applications in biomedical devices, food packaging, and antimicrobial coatings, owing to the strong antibacterial properties of Ag NPs and their compatibility with polymeric matrices. Various preparation approaches have been employed for the development of such nanocomposites, including melt mixing, solution casting, and other conventional polymer processing techniques [27,28,29,30,31,32]. In our previous work, PLA/Ag nanocomposites were successfully synthesized via in situ ring-opening polymerization (ROP), which promoted strong interfacial interactions between the polymer and the Ag nanofillers. This resulted in improved dispersion and enhanced functional properties, including significantly higher flexibility and increased electrical conductivity [33].

Although cast film extrusion is a widely used industrial technique, studies describing the development of PLA-based nanocomposite films using this method remain limited. For instance, Ghanbari et al. utilized a sheet extrusion line to develop biodegradable PLA/poly(butylene succinate-co-adipate) (PBSA)/lignin films, reporting homogeneous lignin dispersion and enhanced ductility with PBSA incorporation, although these improvements were accompanied by reductions in tensile strength and stiffness [34]. Similarly, Khajavi et al. developed antimicrobial PLA-based nanocomposite films reinforced with graphene oxide (GO) nanosheets, reporting improved flexibility, oxygen and UV barrier properties, thermal stability, and antimicrobial activity, which enhanced the preservation of pasteurized milk [35]. In another approach, Fadhel et al. investigated the application of chitosan–clay-based active coatings onto cast-extruded PLA films to significantly enhance their oxygen barrier performance [36]. Furthermore, Bovi et al. prepared PLA/bacterial nanocellulose (BNC) masterbatches via solvent casting prior to cast extrusion, achieving improved BNC dispersion within the PLA matrix and enhanced optical, thermal, and barrier properties, although no significant improvement in mechanical performance was observed [37]. However, while some studies have focused on carbon-based materials, organic fillers, bio-fillers, or surface coatings, to the best of our knowledge, the preparation of PLA nanocomposites based on metal NPs prepared by cast film extrusion has not been reported.

Therefore, this study focused on the development of PLA nanocomposite films containing Ag NPs at 0.5, 1.0, 2.5 and 5.0 wt%. To the best of our knowledge, this is the first study to investigate PLA nanocomposite cast film sheets based on metal NPs, specifically using Ag NPs. A systematic evaluation was conducted on the effect of Ag NP content on their multifunctional properties. The nanocomposites were characterized by a variety of techniques, including infrared spectroscopy, thermal analysis, and mechanical testing, providing detailed insights into their overall material behavior. The aim of this work was to evaluate the effect of Ag NP content on the multifunctional properties of PLA and to investigate the structure-to-property relationship of the resulting materials for potential advanced industrial applications.

## 2. Results and Discussion

### 2.1. Synthesis and Structural Investigation of PLA Nanocomposite Cast Film Samples

**Figure 1:**
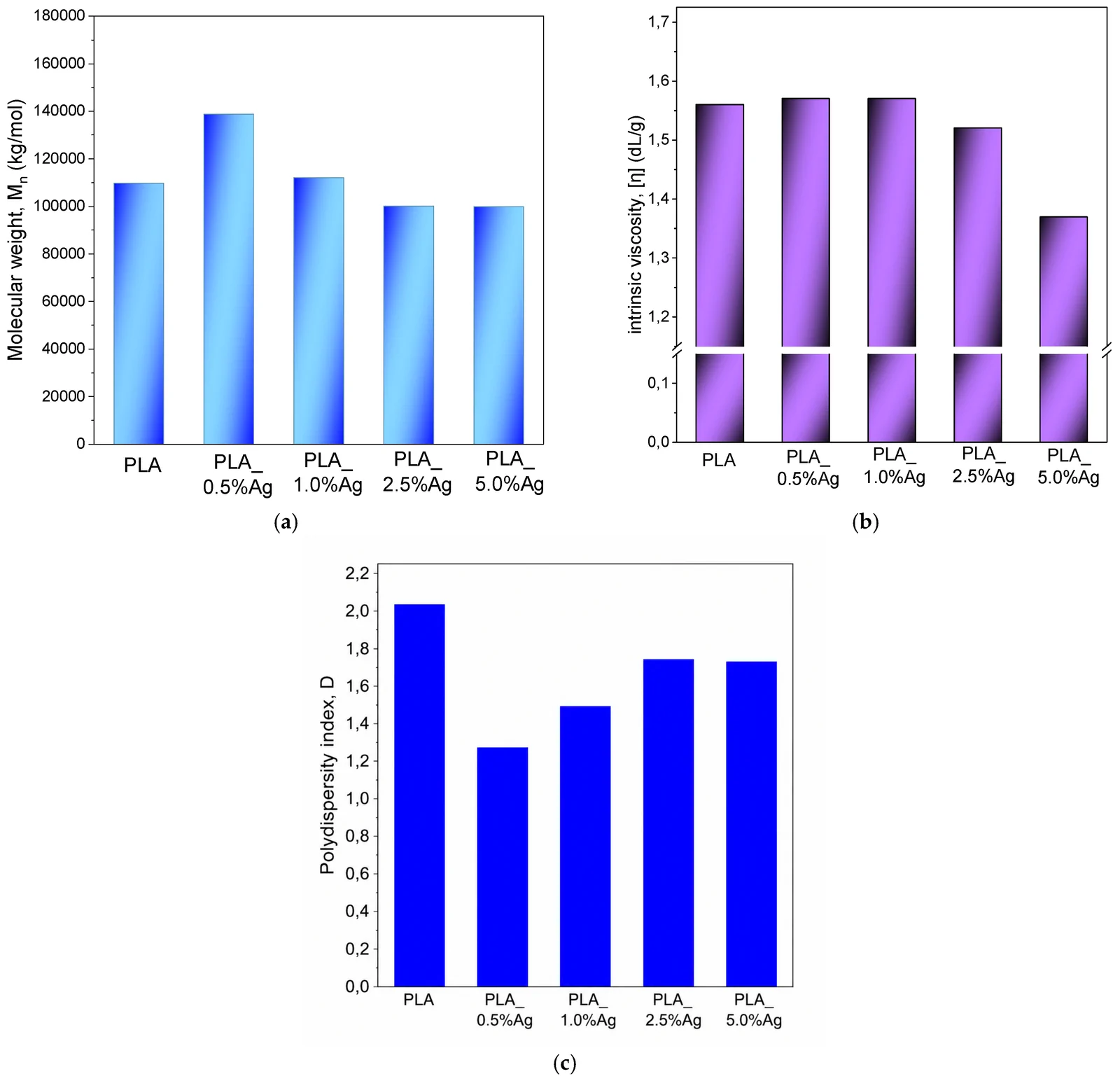
(**a**) Number average molecular weight ($\bar{M_{n}}$) (GPC), (**b**) intrinsic viscosity [η] and (**c**) polydispersity index (Đ) of PLA cast film sample and PLA/Ag nanocomposites. PLA prior to cast film extrusion; ($\bar{M_{n}}$) = 140 Κg/mol, Đ = 1.17 and [η] = 1.64 dL/g.

Gel permeation chromatography (GPC) analysis was performed to determine the number average molecular weight ($\bar{M_{n}}$) and polydispersity (Đ) of PLA and PLA/Ag nanocomposites prepared via cast film extrusion. In parallel, intrinsic viscosity [η] measurements were also conducted (Table S1, Figure 1).

GPC analysis showed that PLA cast film samples exhibited a significantly lower $\bar{M_{n}}$ compared to the unprocessed PLA ($\bar{M_{n}}$ = 109.8 Kg/mol vs. 140 Kg/mol), along with an increase in dispersity. This reduction in $\bar{M_{n}}$, accompanied by a decrease in the intrinsic viscosity, indicated thermomechanical degradation of the polymer chains during processing. It is worth noting that the extrusion process was carried out at a screw speed of 60 rpm, which is twice the screw speed typically used in previous work (30 rpm) [33], in order to promote more effective mixing. The relatively high screw speed may have contributed to the observed molecular weight reduction by increasing the thermomechanical degradation of the samples during extrusion. A similar relationship between molecular weight reduction, chain scission, and decreased intrinsic viscosity has been reported for PLA by Behera et al. following autoclave treatment, where hydrolytic cleavage of ester bonds in the polymer backbone led to chain scission and the formation of shorter polymer chains, resulting in reduced molecular weight and intrinsic viscosity [38]. Although their processing conditions differ from those employed in the present study, their findings support the observed relationship between polymer chain scission, molecular weight reduction, and viscosity loss. It should be noted that the reduction in $\bar{M_{n}}$ is not proportionally reflected by a decrease in intrinsic viscosity, since intrinsic viscosity is influenced not only by $\bar{M_{n}}$ but also by the overall molecular weight distribution. Therefore, an increase in polydispersity (Figure 1c) may partially compensate for the reduction in $\bar{M_{n}}$ through the contribution of higher-molecular-weight fractions. However, at higher loadings, the viscosity of the samples decreased (Figure 1b), indicating that polymer chain diffusion was enhanced in this case. This is consistent with segmental mobility studies, which will be presented later, where the dielectric T_g_ was reduced, also suggesting a decrease in macromolecular chain entanglements [39]. Interestingly, the $\bar{M_{n}}$ of the nanocomposites increased at low loadings of Ag NPs. In particular, $\bar{M_{n}}$ increased from 109.8 Kg/mol for the neat PLA cast film sample to 138.8 Kg/mol for the PLA containing 0.5 wt% Ag NPs (Table S1), indicating that a low concentration of Ag NPs may contribute to preserving the molecular weight of PLA during melt processing. The improvement in molecular weight in the presence of Ag NPs suggested that interactions between the NPs and the PLA matrix may have influenced the degradation processes occurring during cast film extrusion. In particular, Ag NPs may have interacted with reactive species generated under thermomechanical processing conditions, potentially limiting radical-mediated oxidative degradation pathways and reducing polymer chain scission. A similar protective effect was proposed by Râpă et al. for PLA/collagen/AgNP bionanocomposites, where interactions between Ag NPs and free radicals were suggested to contribute to the oxidative stabilization of the polymer matrix [40]. Although the composition and processing conditions of those materials differed from those of the present PLA/Ag nanocomposites, these findings supported the proposed role of the interaction between NPs and the polymer matrix in the improved molecular weight ($\bar{M_{n}}$ and [η]) observed at low Ag NP concentrations.

As the Ag NP content increased beyond 0.5 wt%, the molecular weight gradually decreased, although it remained higher than that of neat extruded PLA, even at 5.0 wt% Ag NPs. This concentration-dependent behavior suggested that the influence of Ag NPs on molecular weight retention became less pronounced at higher NP loadings. In addition to the proposed interactions between Ag NPs and reactive species, changes in the local thermal environment during processing may also have contributed to the observed differences. Nevertheless, the GPC results demonstrated improved molecular weight in the PLA/Ag nanocomposites compared with neat extruded PLA, while the specific contribution of interactions between NPs and the polymer matrix remained a proposed mechanistic explanation rather than a directly established mechanism [41].

**Figure 2:**
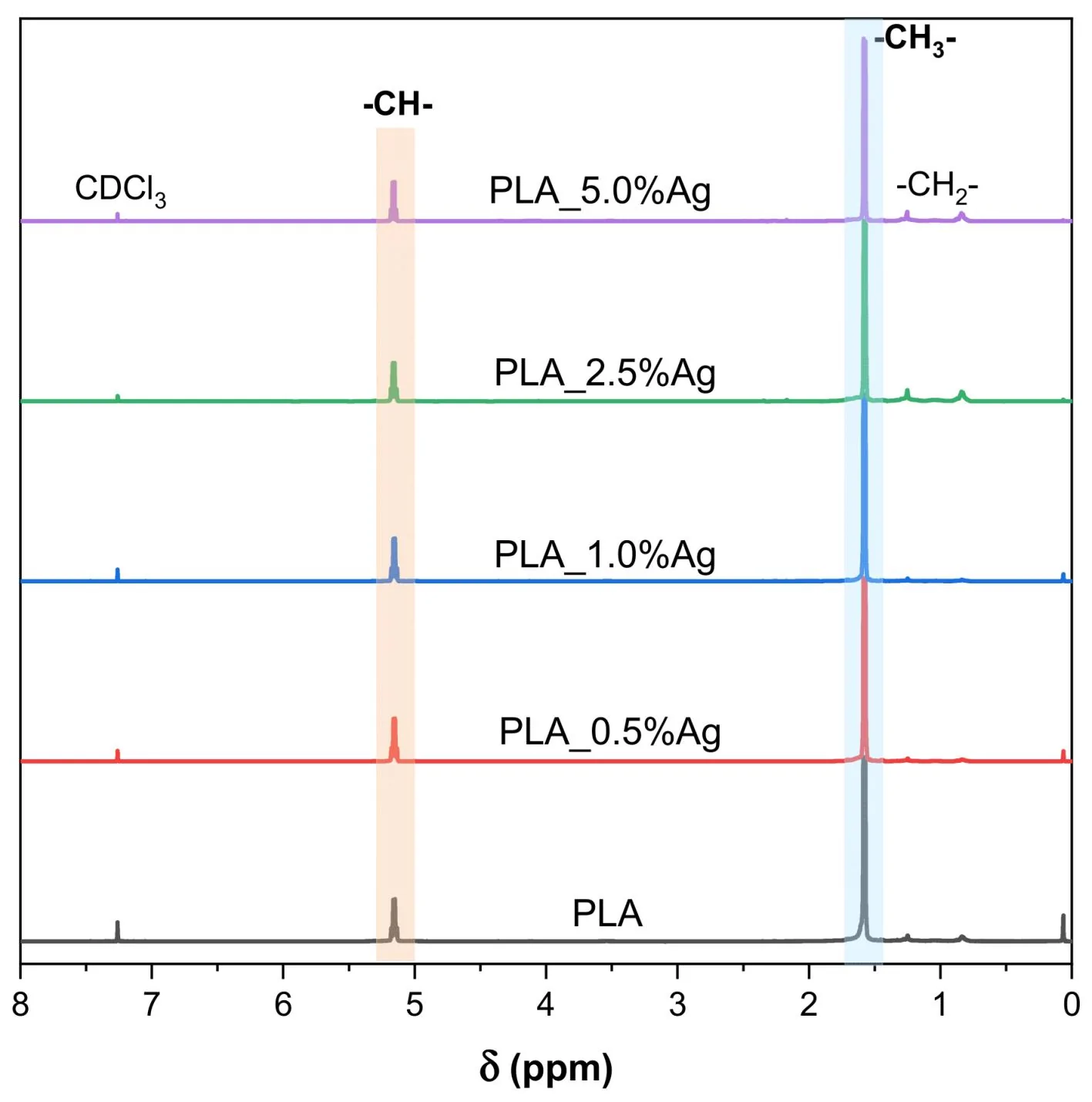
^1^H NMR spectra of the PLA and PLA/Ag nanocomposite samples.

NMR was employed to confirm the successful synthesis of the PLA/Ag nanocomposites, providing structural information on the PLA and Ag NPs by assigning chemical shifts to specific protons. The proposed PLA structure is depicted in Figure 2. Specifically, the ^1^H NMR spectrum of pristine PLA displays two characteristic signals: a multiplet at δ = 5.2 ppm, which corresponds to the methine protons (-CH-), and a sharp peak at δ = 1.8 ppm, corresponding to the methyl protons (-CH_3_-) [42]. As illustrated in the overlaid spectra, the incorporation of Ag NPs did not induce any significant shifts or the appearance of new peaks in the polymer’s core regions. The absence of significant changes in the chemical shifts indicated that the chemical environment of the PLA backbone was largely preserved following the incorporation of Ag NPs, suggesting that no major changes occurred in the covalent structure of the polymer.

**Figure 3:**
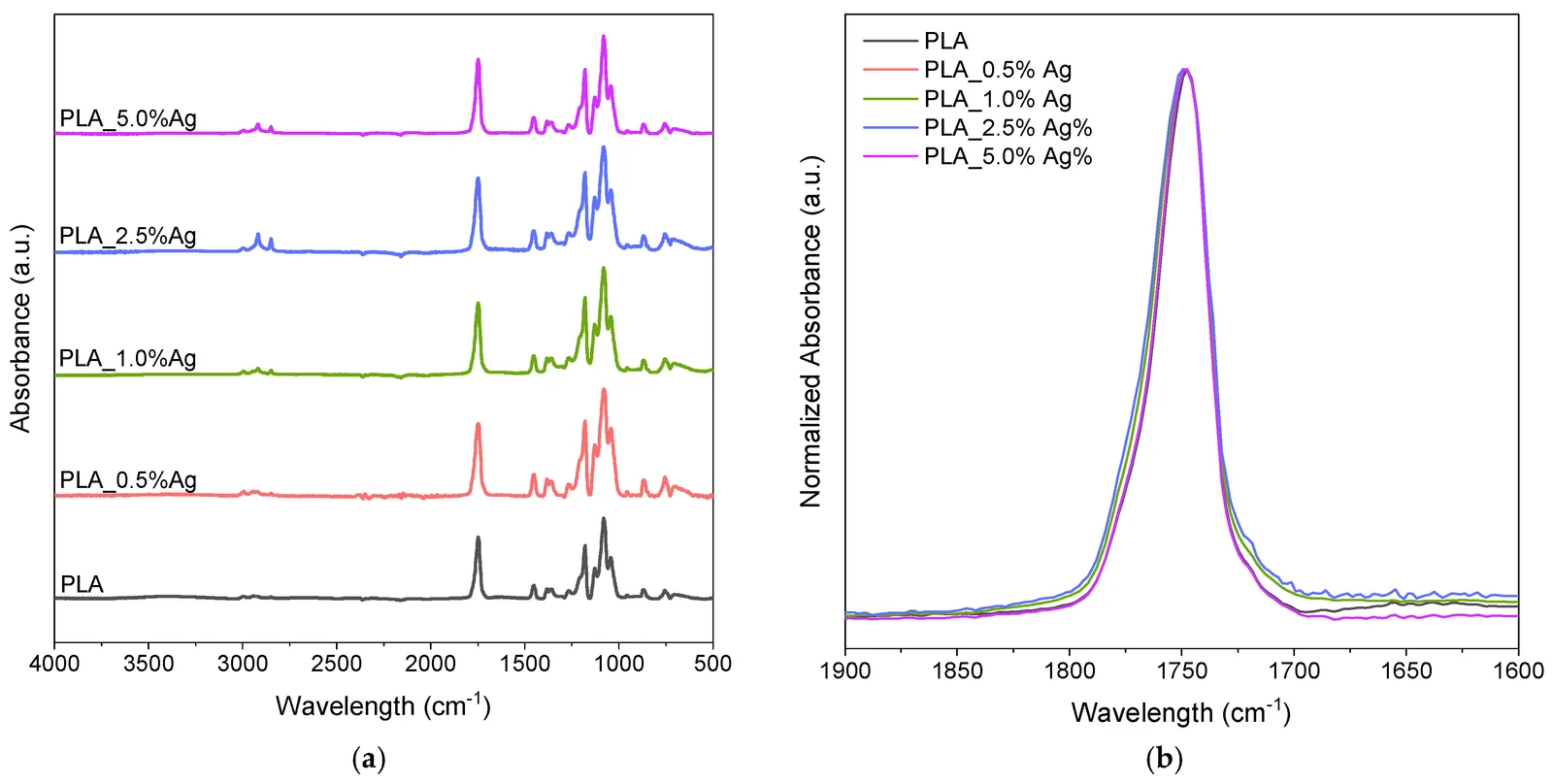
(**a**) ATR-FTIR spectra of PLA cast film and PLA/Ag nanocomposites and (**b**) zoomed-in view area of normalized spectra in carbonyl region.

Figure 3a shows the ATR-FTIR spectra of the PLA cast film and PLA/Ag nanocomposites. The characteristic peaks of PLA are observed at 3000–2850 cm^−1^, corresponding to symmetric and asymmetric C-H stretching vibrations; at 1750 cm^−1^, they are attributed to carbonyl (C=O) stretching; and those in the regions 1500–1400 cm^−1^ and 1100–1000 cm^−1^ are associated with C-H bending and C-O stretching vibrations, respectively [42,43]. The incorporation of Ag NPs did not lead to the appearance of new absorption bands, nor to noticeable shifts or intensity changes of the characteristic PLA peaks, indicating the absence of chemical interactions between the polymer matrix and the Ag NPs [44,45].

Focusing on the carbonyl region around 1750 cm^−1^ (Figure 3b), minor changes in the peak intensity were observed for the PLA/Ag nanocomposites compared to neat PLA, without any shift in the peak position. These slight intensity variations suggested weak interfacial interactions between the Ag NPs and the PLA matrix, which were not strong enough to cause a noticeable change in the peak of carbonyl groups. A similar behavior was also observed in our previous study [33], where the carbonyl region remained unaffected by the presence of Ag, with only the sample containing 1 wt% Ag incorporated in situ during the ring-opening polymerization (ROP) of L-lactide showing noticeable changes in the C=O region, suggesting possible interfacial interactions between the Ag NPs and the PLA matrix. Overall, the FTIR results suggested that, if present, the interaction between the Ag NPs and the PLA matrix was weak and did not result in a significant change in the carbonyl groups.

**Figure 4:**
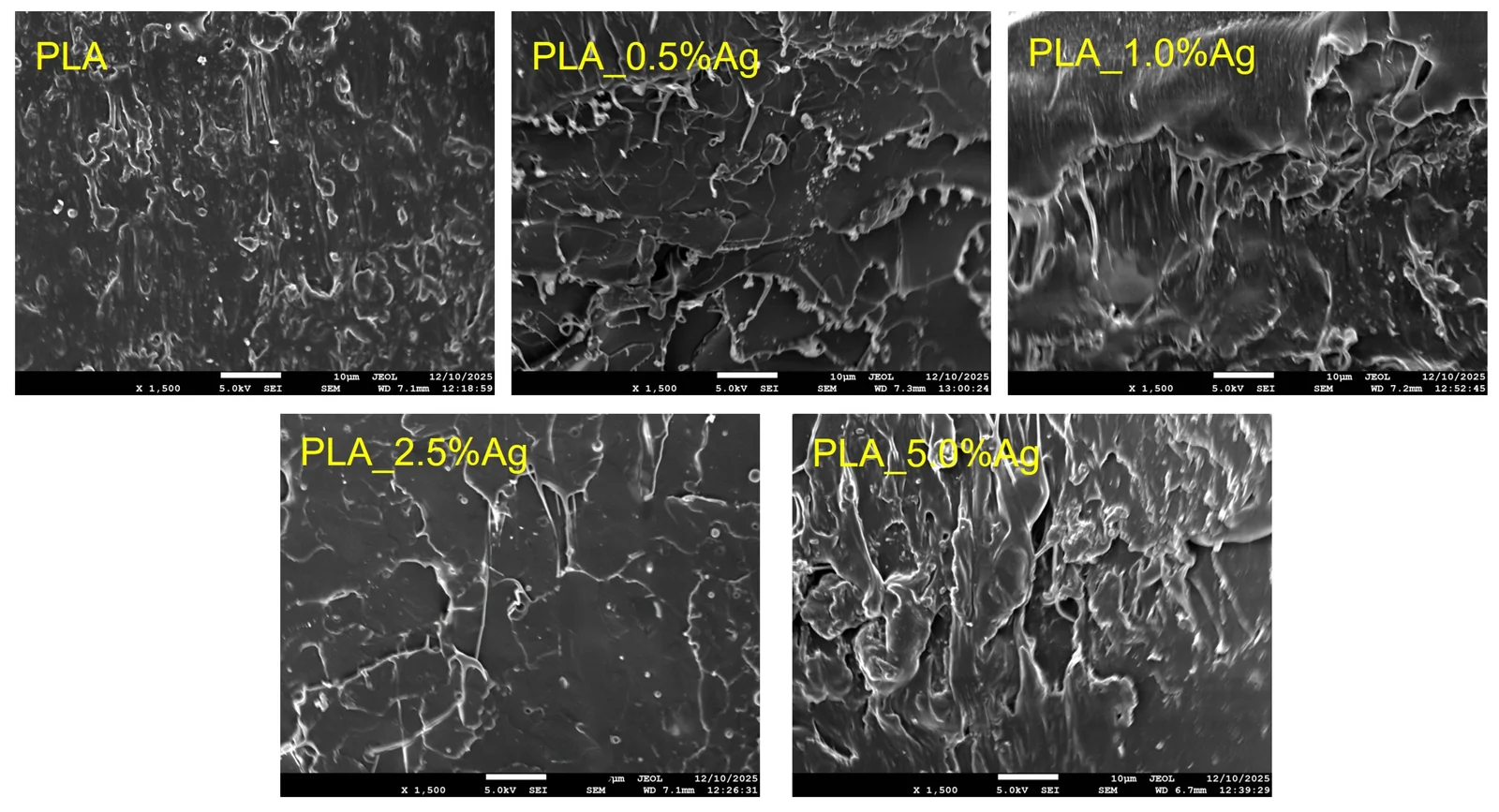
SEM images of cast film samples.

The morphological study of the cast film samples, as obtained from SEM analysis, is presented in Figure 4. Neat PLA exhibited a relatively homogeneous morphology along with a specific direction, which can be attributed to the sample preparation process via cast extrusion, a method that imposes tensile stresses on the melt as it passes through the die [46]. The melt flow during extrusion led to stretching and partial orientation of the PLA macromolecular chains along the flow direction, as previously reported by Tcharkhtchi et al. for an extruded PLA/PBS blend containing 80 wt% PLA and 20 wt% PBS, which were produced by extrusion and subsequent drawing through cooling rolls. This flow-induced orientation was reflected in the SEM images through the appearance of linear and elongated structures aligned parallel to the casting flow direction [47].

In contrast, the cast samples containing Ag NPs exhibited a noticeable change in cross-section morphology, characterized by increased heterogeneity compared to neat PLA. This observation suggested that the presence of NPs influenced the microstructure of the polymer matrix. This behavior was consistent with the literature on polymer nanocomposites processed under flow conditions, thereby influencing the orientation and crystallization behavior of macromolecular chains. Recent findings by Eriksson et al. demonstrated that NPs function as nucleating agents and can influence flow-induced crystallization in polymer nanocomposites by providing heterogeneous nucleation sites that promote crystallite formation during cooling, which may contribute to the development of a more structured polymer morphology [48]. In alignment with these manufacturing-driven effects, Soleymani Eil Bakhtiari et al. highlighted that the processing sequence directly influenced structural homogeneity and phase interactions in reactively compatibilized polymer blends, with the two-step blending approach promoting more uniform phase dispersion and stronger interfacial adhesion, which ultimately resulted in improved tensile strength and elongation compared with single-step blending [49]. It should be noted that the morphological heterogeneity observed in the SEM cross-sectional images indicated changes in the PLA matrix associated with Ag NP incorporation, rather than providing direct evidence of the three-dimensional distribution or the dispersion of the Ag NPs. The importance of filler distribution and orientation in determining the properties of polymer composites was also highlighted by Guo et al., who demonstrated that the alignment of carbon fibers relative to the applied electric field significantly influenced electrical conductivity and electromagnetic interference shielding effectiveness. Their findings emphasized the critical role of filler arrangement in establishing structure–property relationships [50].

### 2.2. Thermal Properties, Crystalline Behavior and Molecular Dynamics

**Figure 5:**
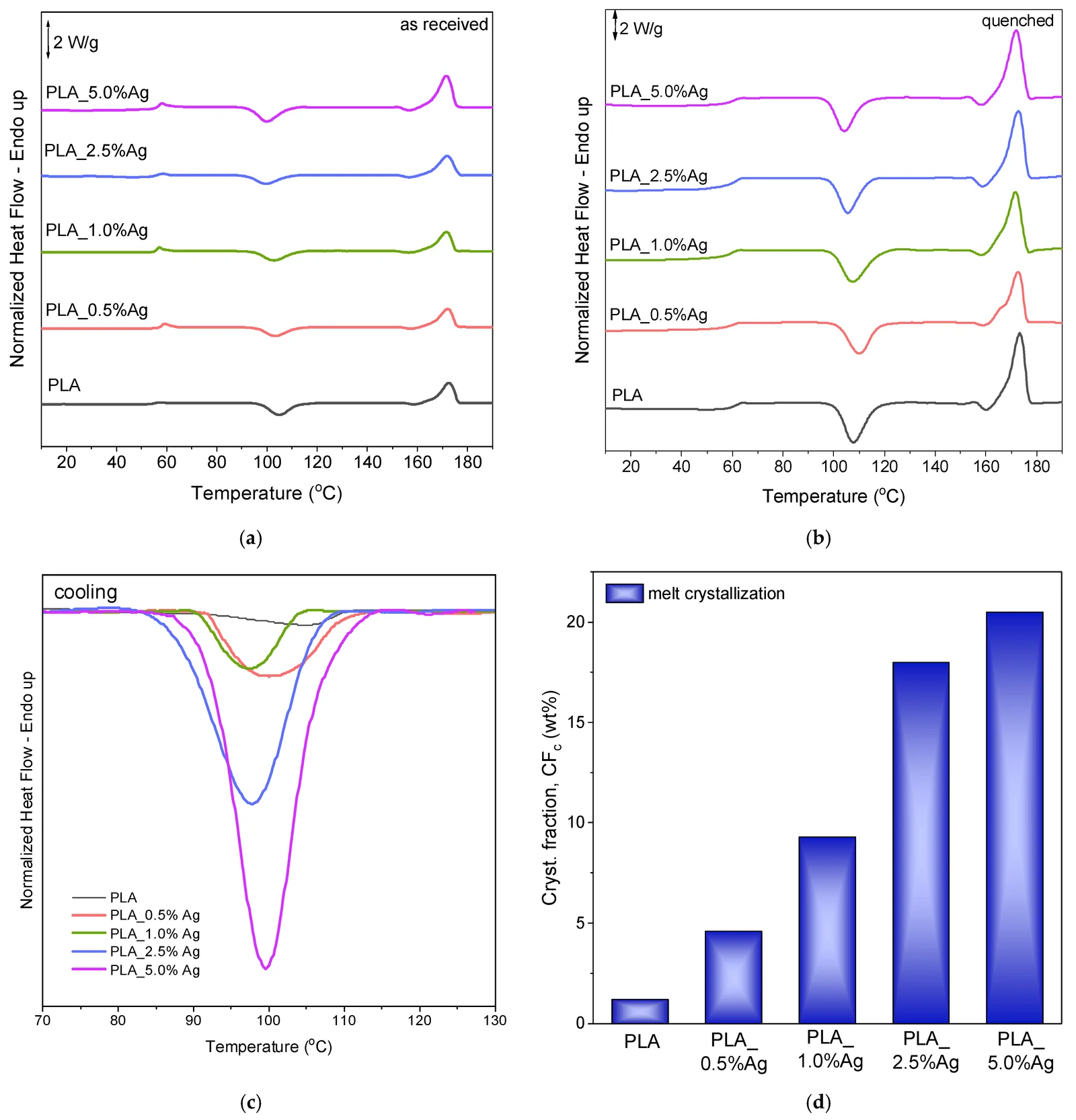
DSC curves of the PLA cast film sample and PLA nanocomposites during (**a**) the first and (**b**) the second heating cycles at 20 °C/min and (**c**) cooling at 10 °C/min, and (**d**) the crystalline fraction (CF_c_) during melt crystallization.

The DSC scans of the cast film samples are presented in the next figure for the first and second heating cycles, as well as traces during slow cooling, in order to evaluate the effect of Ag NPs on the thermal transitions and crystallization behavior of PLA. During both the first and second heating cycles (Figure 5a,b), all samples exhibited a well-defined melting endotherm peak T_m_ at 172 °C [51]. The absence of additional melting peaks suggests the lack of different crystal populations [33]. A defined glass transition step (T_g_) was detected for all samples at temperatures close to 60 °C (Table 1) [52], confirming that the incorporation of Ag NPs did not significantly alter the amorphous phase mobility of PLA [53].

T_g_ was reduced from 60 °C for neat PLA to 59 °C for the PLA/Ag NP samples containing 0.5 and 1.0 wt% Ag NPs; although minor, it may indicate a slight increase in the mobility of the PLA chains, despite the fact that for the PLA Ag NP samples, $\bar{M_{n}}$ was significantly higher compared to the neat PLA (Figure 1a). The Ag NPs may disturb the local packing of PLA chains and create interfacial regions with slightly increased free volume, which did not affect T_g_ here. However, we expected more than one parameter to affect T_g_. For example, the lowering of M_n_ was expected to cause a mild T_g_ decrease, while, at the same time, the existence of NPs and the dense interfacial polymer layer around them was expected to increase T_g_ [54,55]. At the semicrystalline state, additional parameters are involved, with both hindering/enhancing effects on polymer mobility (e.g., constraints and spatial nanoconfinement between crystals) [56,57]. The issue of segmental mobility will be investigated in detail later, along with the BDS findings.

During cooling (Figure 5c), the crystallization temperature of the melt (T_c_) forneat PLA was observed at 106 °C, while a shift towards lower temperatures, in the range of 97–99 °C, was recorded for the PLA/Ag nanocomposites. A similar trend has been reported by Chu et al., who studied PLA/ZnO nanocomposites and observed that the T_c_ values slightly decreased with the addition of NPs. This deceleration of the crystallization rate during cooling is typically attributed to the restriction of polymer chain mobility near the interfaces, even when NPs act as potential heterogeneous nucleation sites [58]. Regarding the crystalline fraction from the melt crystallization peak (CF_c_) (Figure 5d), the incorporation of Ag NPs led to a noticeable increase compared to neat PLA. As shown in Table 1 and Figure 5d, CF_c_ increased progressively with increasing Ag content. This behavior suggests that Ag NPs acted as nucleating agents, promoting nucleation and heterogenous crystallization. This is in good agreement with Demchenko et al., who have reported a similar trend, where the addition of Ag NPs enhanced the crystallization behavior of PLA [59]. In addition, the increased crystallinity can be partially attributed to the reduced molecular weight of PLA (Figure 1), where shorter polymer chains are able to reorganize more easily into ordered crystalline structures [60]. This complex behavior, the shift in T_c_ to lower temperatures alongside the progressive increase in CF_c_, can be strongly correlated with both the reduction in $\bar{M_{n}}$ and [η] and the high NP loading. Specifically, for the 2.5 and 5.0 wt% nanocomposites, the drop in $\bar{M_{n}}$ was more pronounced, allowing the PLA chains to reorganize more easily into ordered crystalline structures. Concurrently, the large population of Ag NPs in these high-loading samples provided an abundance of nucleation sites. Therefore, the synergistic effect of enhanced chain mobility (due to lower $\bar{M_{n}}$ and intensive heterogeneous nucleation) effectively led to the development of a higher crystalline fraction, despite crystallization occurring at lower temperatures. Thus, for high loadings, crystallization (Figure 5d) was affected by the nucleation activity of the Ag NPs and the enhanced chain diffusion of the macromolecular PLA chains, which was caused by the reduction in the molecular weight; these phenomena directly influenced the crystallizability of the samples [61].

Figure S1 presents the variation in (a) the crystallization temperatures and (b) the corresponding crystalline fractions for cold and melt crystallization. As shown in Figure S1a, the cold crystallization temperature (T_cc_) exhibited small changes with the Ag content compared to neat PLA. However, as the Ag content increased up to 5.0%, the molecular weight gradually dropped back down. Correspondingly, the T_cc_ progressively decreased to 104.0 °C. This downward trend indicated that the combination of a lower molecular weight and the intense heterogeneous nucleation effect of the Ag NPs at high loadings allowed the PLA matrix to crystallize much more easily and at significantly lower temperatures. A similar phenomenon was observed by Yang et al., who investigated binary PLA blended systems with varying molecular weights and reported that high-molecular-weight fractions significantly hindered chain movement and reduced the overall relaxation rate, thereby shifting the crystallization process to higher thermal levels [62]. In contrast, the melt crystallization temperature (T_c_) was shifted to lower temperatures relative to neat PLA, indicating that crystallization during slow cooling started at lower temperatures. This variation in T_c_ does not necessarily indicate a reduction in nucleation only, since T_c_ is affected by combined effects such as nucleation and chain mobility. In the present system, the simultaneous increase in CF_c_ suggests that the Ag NPs promoted the overall crystallization process, despite the lower T_c_ [63].

Regarding the crystalline fractions (Figure S1b), CF_c_ increased progressively with Ag content, confirming that Ag NPs promoted crystal formation during cooling by acting as nucleating agents. On the other hand, CF_cc_ showed a more gradual change. This behavior is in agreement with the work of Klonos et al., who investigated the effects of low nanofiller loadings with various dimensional geometries (1D, 2D, and 3D) in poly(ethylene succinate) (PESu)-based nanocomposites. They demonstrated that even at low concentrations, the fine dispersion of NPs provided a continuous network of nucleation sites that synchronized crystal growth, thereby facilitating a more controlled and uniform cold crystallization process through tailored interfacial interactions [63].

**Figure 6:**
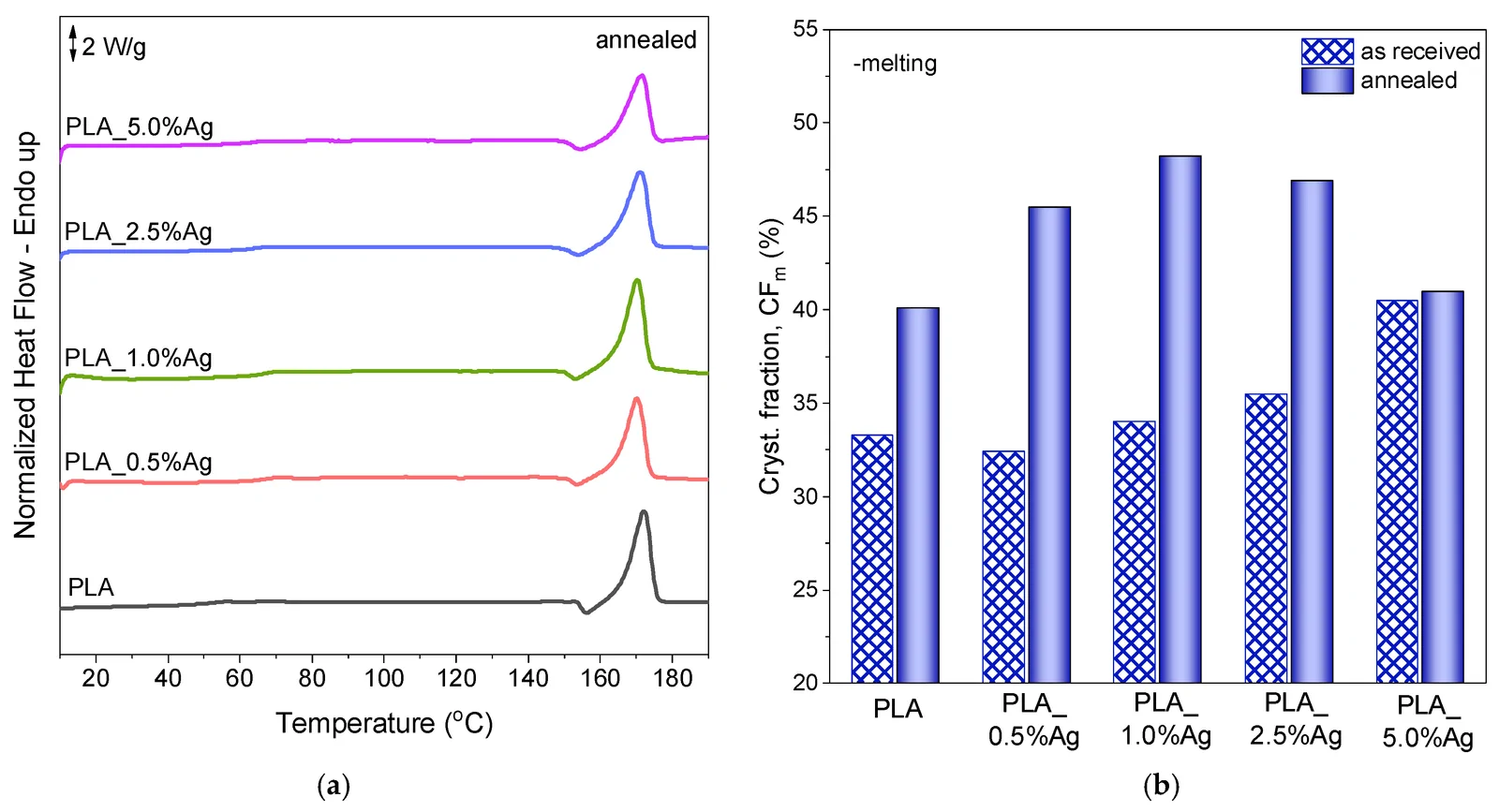
(**a**) DSC curves of the materials after the annealing experiments and (**b**) the crystalline fraction of the melting peak (CF_m_) of the samples before and after annealing.

The thermal transitions of the cast films after annealing at 110 °C for 1 h are shown in Figure 6a. The neat PLA cast film exhibited a main T_m_ at approximately 172 °C. The incorporation of Ag NPs resulted in a slight decrease in T_m_ to around 170–171 °C, while the melting peak shape and width remained similar. The annealed samples exhibited consistently higher crystalline fractions of melting (CF_m_) compared to those obtained during the first heating cycle (Figure 5a), indicating that annealing effectively enhanced the crystallinity of the samples (Figure 6b) [64]. The incorporation of Ag NPs led to higher crystallinity compared to neat PLA, consistent with previous reports that inorganic NPs can act as nucleating agents for PLA [65], and this is in agreement with the trends observed for the crystalline fraction of the melt-crystallized samples (Figure 5d).

**Figure 7:**
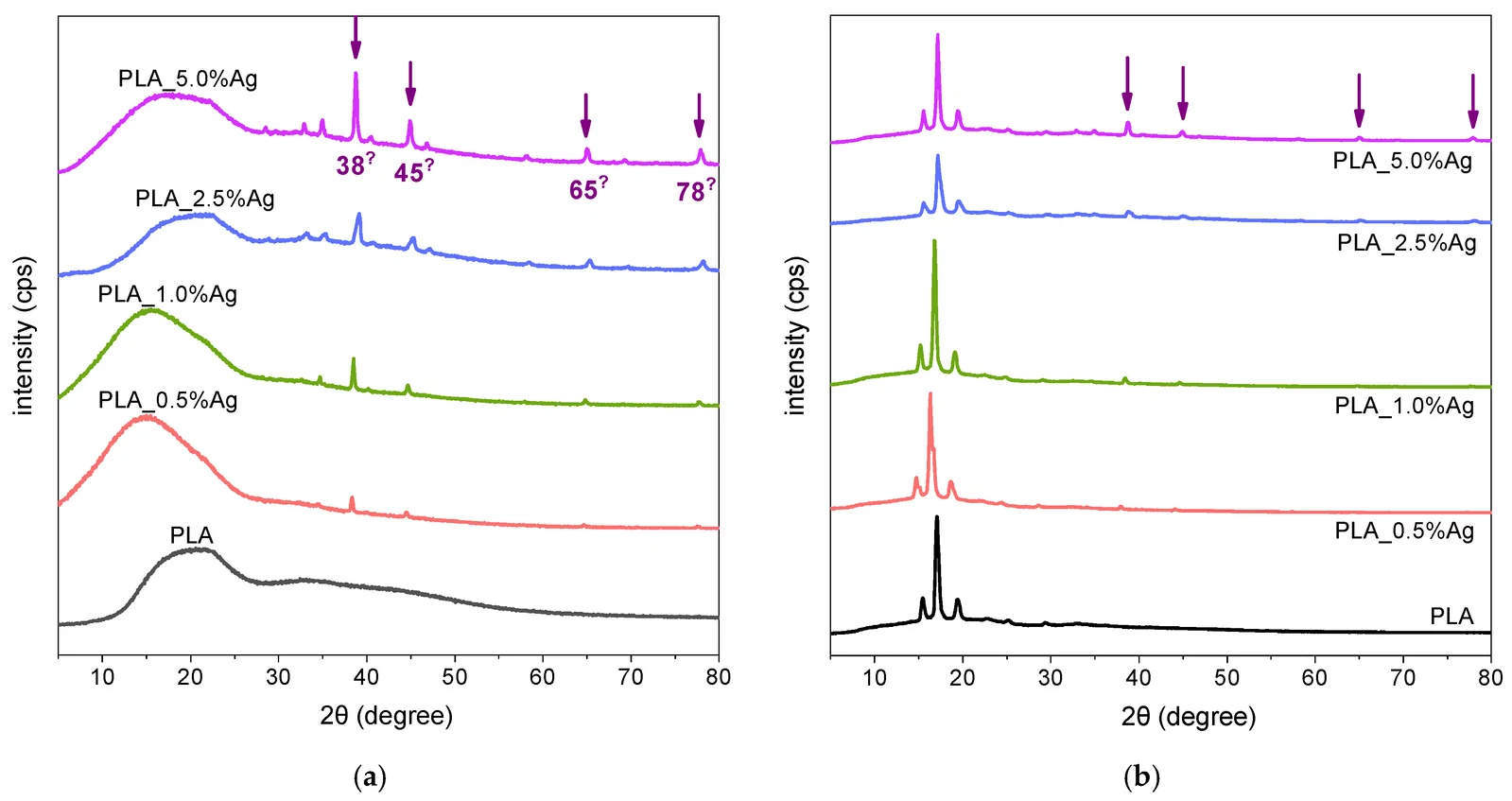
(**a**) The XRD patterns of PLA cast film samples and its nanocomposites and (**b**) the annealed XRD patterns. The arrows signs are just guidance for the eye.

Figure 7 illustrates the XRD patterns of the PLA cast film and PLA/Ag nanocomposites (a) before and (b) after annealing at 110 °C for 1 h. Prior to annealing (Figure 7a), neat PLA exhibited a predominantly amorphous structure, as evidenced by the presence of a broad diffraction halo centered at approximately 10–25° [66]. Upon the incorporation of Ag NPs, additional diffraction peaks appeared at 2θ around 38°, 45°, 65°, and 78° (arrows signs), which are in agreement with previously reported results in the literature [27,67]. The intensity of the Ag-related peaks increased with increasing Ag content in the PLA matrix, indicating the successful incorporation and effective dispersion of Ag NPs within the polymer. These results suggests that the NPs were distributed both on the surface and within the bulk of the PLA cast film [67]. Regarding PLA, no additional diffractions peaks appeared for the nanocomposite samples. Furthermore, no distinct diffraction peaks corresponding to crystalline silver oxides, particularly Ag_2_O at 32.8° and 54.9°, were observed, suggesting that the Ag NPs predominantly retained their metallic crystalline structure after cast film extrusion. After annealing (Figure 7b), PLA exhibited the expected crystalline diffraction behavior at approximately 15°, 17°, and 19°, corresponding to its crystalline phases [68]. Concerning the nanocomposites, the incorporation of Ag did not alter the crystalline diffraction peaks of PLA. The diffraction peaks corresponding to Ag were still observed after annealing at approximately 38°, 45°, 65°, and 78° [69].

For polymeric systems, crystallization strongly affects the structure and final properties of the material. Isothermal crystallization studies can show how the type of NPs and their interactions with PLA influence mechanical and thermal behavior. This information is important for optimizing materials for specific applications [70,71].

**Figure 8:**
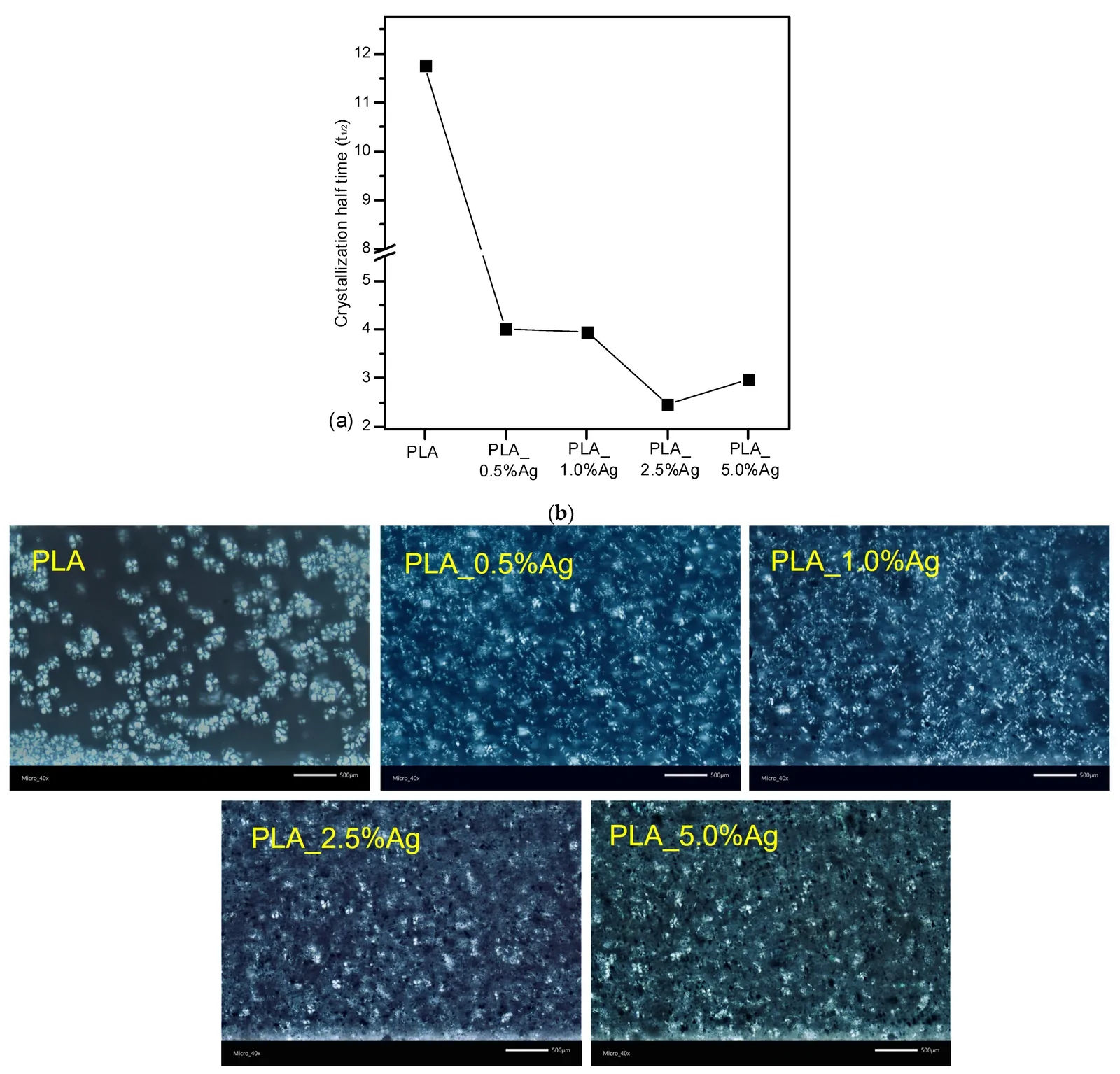
(**a**) The crystallization half-time of the materials, and (**b**) PLΜ images after isothermal melt crystallization at 120 °C of the cast film samples (scale bar: 500 μm).

Preliminary isothermal melt crystallization of neat PLA and PLA nanocomposites was examined via DSC. The corresponding crystallization exotherms are shown in Figure S2a. The relative degree of crystallinity, X(t), was determined using the equation provided below in order to evaluate the crystallization rate of the materials. The evolution of X(t) with crystallization time is presented in Figure S2b, assuming a linear relationship between crystallinity development and heat release during crystallization.

(1) $$X(t)=\frac{\int_{0}^{t}(dH_{c}/\mathrm{dt})\mathrm{dt}}{\int_{0}^{\infty }(dH_{c}/\mathrm{dt})\mathrm{dt}}$$

where dH_c_ denotes the enthalpy of crystallization of a really small fraction of time interval dt. The limits t and ∞ on the integrals indicate the elapsed time during the process of crystallization and at the end of crystallization, respectively. The crystallization half-time (τ_1/2_) indicates the necessary time required to reach 50% of overall crystallinity during the isothermal melt crystallization process. Then, τ_1/2_ data were calculated (Figure 8a) in order to estimate the crystallization rate of the materials indirectly [70,72]. The experiments were carried out at 120 °C. The subsequent heating traces were also recorded (Figure S2c).

Figure 8a presents the τ_1/2_ data of the cast film samples. The presence of Ag NPs significantly affected the crystallization behavior of materials, decreasing the τ_1/2_ of PLA by more than 65%. This rapid reduction aligns with the thermal transitions of the PLA Ag NPs, where the crystalline fraction increased during slow cooling (melt crystallization, Figure S1b), and the Ag promoted the crystallization rate of the samples. The maximum crystallization rate was achieved at 2.5 wt% loadings (i.e., lowest τ_1/2_), whereas at higher concentrations Ag NPs formed agglomerations, slightly reducing the crystallizability of the sample. Similar behavior was observed for PLA nanocomposites containing Ag NPs, prepared by melt mixing [33]. The subsequent heating traces (Figure S2c) revealed that the materials exhibited typical multiple melting peaks associated with different crystallization populations [73]. It is important to note that a second melting peak shoulder (III) was observed, indicating improved thermal stability for the nanocomposite samples.

The PLM results for the neat PLA and PLA/Ag nanocomposite cast films are presented in Figure 8b. Compared with neat PLA, the incorporation of Ag NPs resulted in a higher nucleation density and the formation of a larger number of smaller spherulites. These observations indicate enhanced heterogeneous nucleation, supporting the data derived from the isothermal crystallization kinetic analysis (Figure 8a). This behavior can be attributed to the role of Ag NPs as effective heterogeneous nucleating agents, which provide additional nucleation sites and promote the formation of uniform distributed crystalline structures [64,74]. Similar behavior, involving the formation of a denser network of smaller spherulites in the presence of various NPs, has been reported for PLA-based nanocomposites (Tarani et al.) [75].

We now focus on the effects on molecular dynamics, with emphasis on segmental mobility. First, it should be noted that based on the BDS results, none of the nanocomposites exhibited electrical conductivity despite the presence of the metal NPs. This suggests the absence of continuous Ag paths, at least for electrical percolation. The presence of Ag within the polymer matrix led to a slight elevation in the dielectric permittivity, e.g., at room temperature from ~3 (PLA) to ~4.5 (PLA_2.5% Ag).

**Figure 9:**
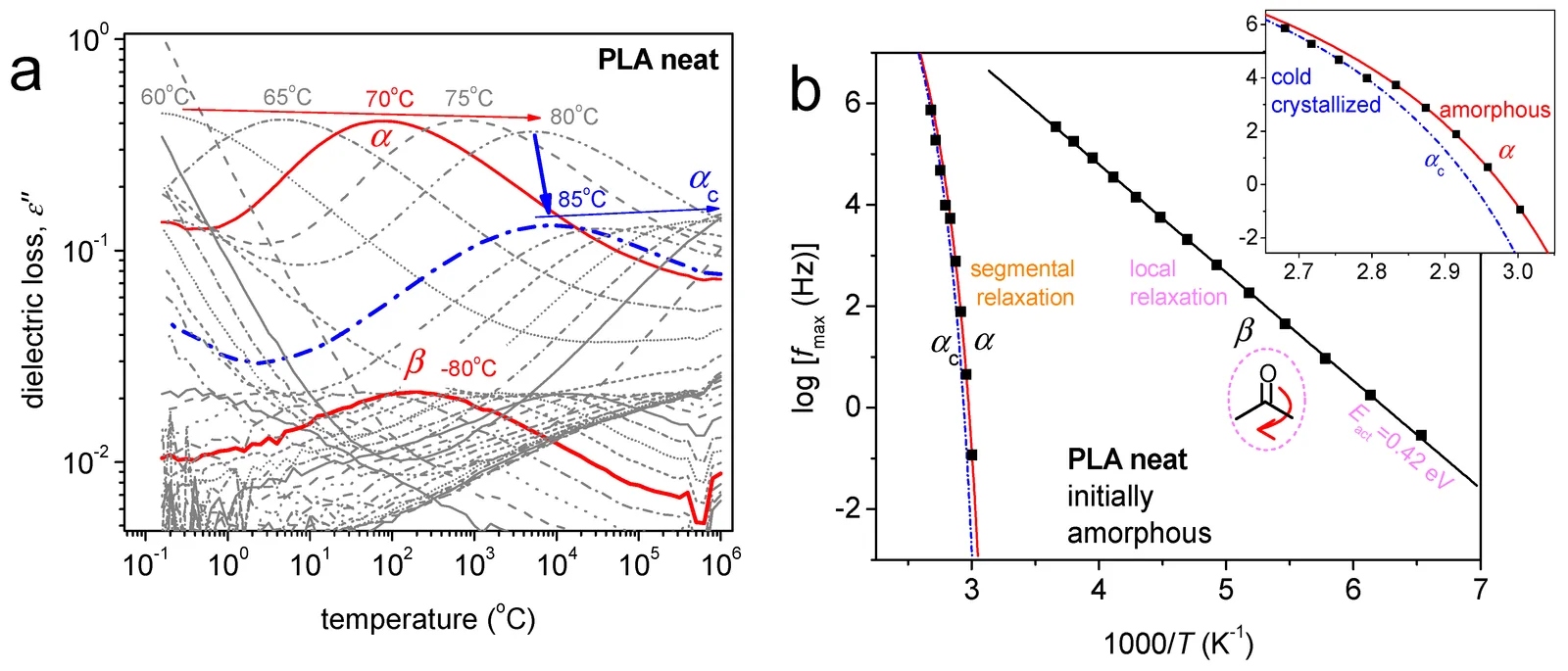
(**a**) Raw BDS data for neat PLA, namely isothermal curves of ε″ as a function of frequency for the various temperatures. Indicated local and segmental relaxations are recorded. (**b**) A dielectric relaxation map (Arrhenius plots) for neat PLA, depicting the time scale of β, α and α_c_ relaxations.

A widely employed method for investigating molecular mobility regarding polymeric systems is broadband dielectric spectroscopy [76]. Briefly, the local and segmental mobilities of local molecular sites and the overall cooperative motion of the polymer chains can be indirectly screened via the corresponding dipolar motions. In this context, BDS allows for evaluation of the temperature and frequency dependence of dielectric permittivity, in particular the imaginary part (ε″—dielectric loss) of said motions, recorded as peaks of ε′′. In Figure 9a, the raw BDS data of PLA are shown. Therein, at T < T_g_, the local β relaxation is recorded. Β relaxation originated from the crankshaft rotation of the ester group (-C=O) at the backbone of PLA [77]. When T approached and exceeded T_g_, the dielectric signal was stronger, and the main α relaxation entered the frequency window. α relaxation was the dielectric analogue of the glass transition [76]. At T~80–85 °C, the implementation of cold crystallization induced strong suppression of α and a transition to the so-called α_c_ relaxation [78,79]. At T >> T_g_ and for the lower frequencies, ionic conductivity effects governed the dielectric signal, which increased by orders of magnitude.

Employing known methods of analysis of BDS spectra in terms of model functions [80], a summarizing dielectric relaxation map was created for neat PLA and is presented in Figure 9b. Here, the expected linear time scale (Arrhenius behavior) is depicted for local β relaxation with a T-independent activation energy of E_act_~0.4 eV. On the other hand, the amorphous α relaxation and the retarded crystallization-affected α_c_ relaxation exhibit ‘curved’ time scales (inset to Figure 9b). The latter is characteristic of ‘cooperative’ segmental mobility [53].

**Figure 10:**
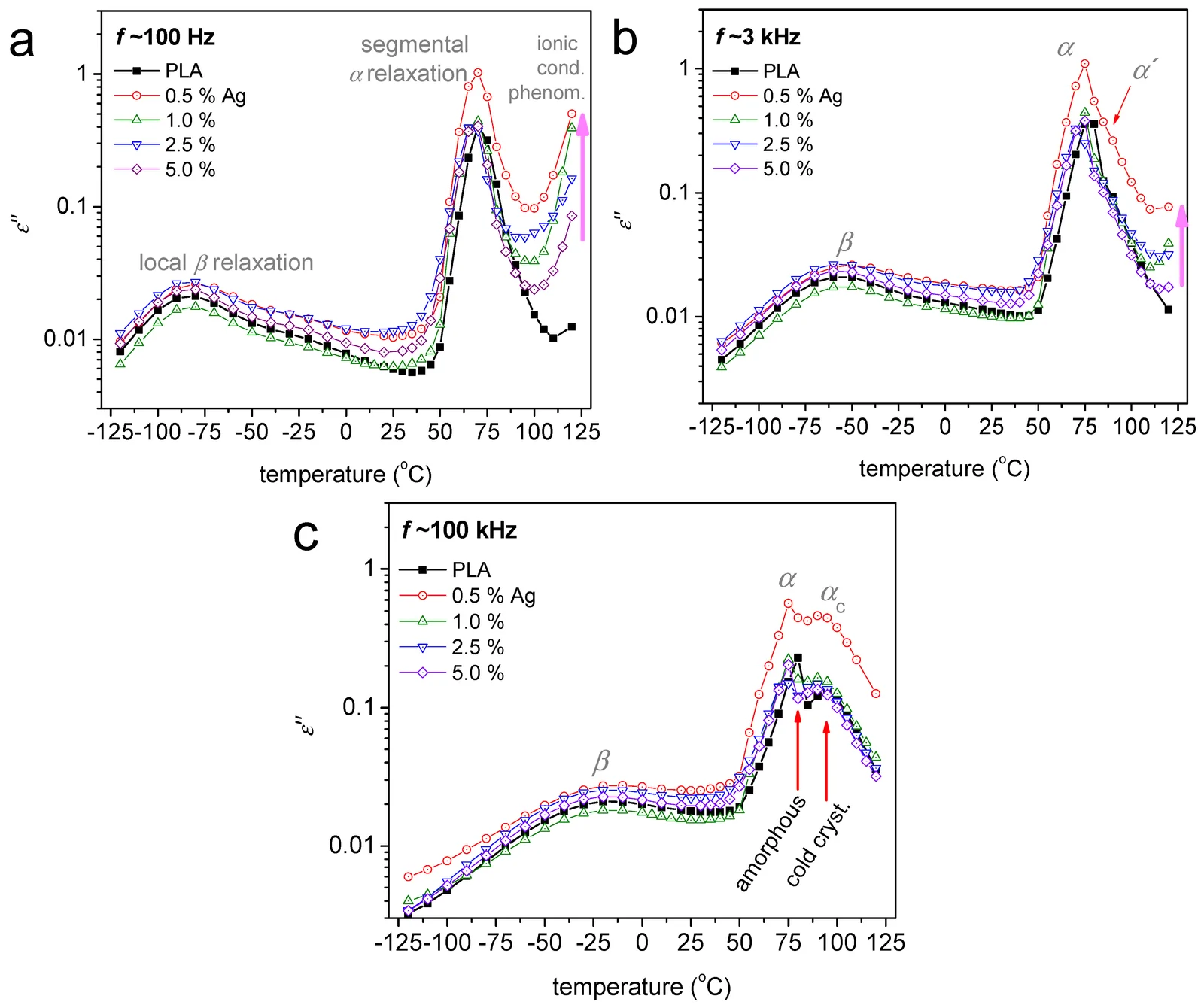
Comparative isochronal BDS traces of ε″(T). The data are shown at three selected f values of (**a**) 100 Hz, (**b**) 3 kHz and (**c**) 100 kHz. The local β, segmental α/α_C_ and segmental-like α′ relaxations are indicated on the plots.

Regarding the nanocomposites, the BDS data are presented along with those of neat PLA in the more reader-friendly way of isochronal plots, i.e., ε″ against T for selected frequencies, as shown in Figure 10a–c. These images depict the advantages of a more direct comparison with the DSC thermograms as well as the strong, intermediate and low involvement of ionic conductivity effects at low (Figure 10a), intermediate (Figure 10b) and high frequencies (Figure 10c).

In Figure 10a, the nanocomposites qualitatively demonstrate the same number of relaxations (peaks), namely β and α. However, at the higher T, the effects of ionic phenomena are enhanced compared to neat PLA. This is due to the combination of the ab initio existence of the conductive Ag NPs, the increased interfacial polarization (either of the NPs or the polymer crystals), and the easier transport of ions throughout the PLA matrix [81,82]. The latter is compatible with the aforementioned assumption of increased free volume in the nanocomposites. In Figure 10b, in the region of segmental dynamics, there seems to exist an addition contribution recorded as a weak shoulder to α, named here as α′ relaxation. Upon analysis, this relaxation is clearly resolved for the nanocomposites with 0.5, 2.5 and 5.0 wt% Ag NPs.

**Figure 11:**
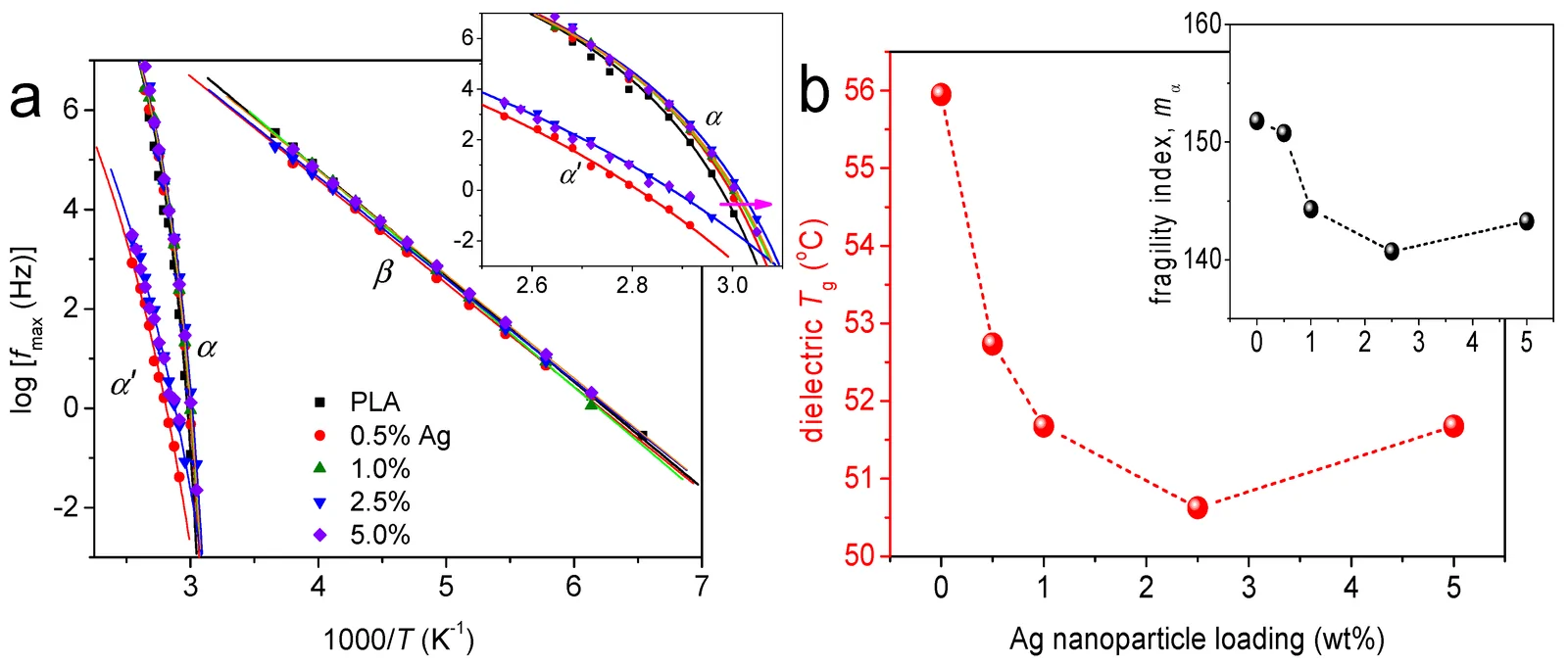
(**a**) A full-scale dielectric relaxation map of PLA and the PLA/Ag. The curved and straight lines crossing the experimental data are fittings of the Vogel–Fulcher–Tammann–Hesse and Arrhenius equations, respectively. The inset to (**a**) shows a focus on the segmental dynamics range. (**b**) The effects of Ag loading on the (main figure) dielectric T_g_ and (inset to **b**) on the fragility index of the α relaxation, m_a_.

In the dielectric map that follows Figure 11a, α′ exhibits a cooperative-like time scale behavior, like that of α. In addition, by extrapolating its time scale to the equivalent frequency of calorimetry, α′ tends to merge with α, or else, crosses the T_g_. Combining these observations with the fact that α′ was recorded in the amorphous state of the polymer (i.e., prior the evolution of cold crystallization), the relaxation seems to originate from the retarded segmental dynamics of the polymer chains located at the nanometric interfacial PLA/NP zone [80,82,83].

In the map of Figure 11a, the local β relaxation is mainly unaffected in the nanocomposites. This was more or less expected. The addition of Ag NPs led to weak acceleration of α relaxation (inset to Figure 11a) and, subsequently, to a lower equivalent dielectric T_g_. Based on further analysis of the dielectric map of α, we estimated the ‘dielectric T_g_’. This was found to be equal to 56 ^ο^C for PLA and to monotonically drop by 3–5 K for the nanocomposites with 0.5–2.5 wt% Ag NPs, as depicted in Figure 11b. Finally, it increased by 1K for 5.0 wt% Ag. From the ‘curvature’ of the α relaxation’s time scale, the kinetic fragility index, m_α_, was calculated [84]. The values are quite high (in the order of 140–150), as shown in the inset to Figure 11b. The Ag loading dependence of m_α_ is qualitatively similar to that of the dielectric T_g_, i.e., mainly exhibiting a monotonic drop. For relatively simple polymeric systems [85], this index can be used as a measure of the ‘cooperativity degree’ [53]. A T_g_ drop accompanied by an m_α_ drop has been reported within similar systems [86] and rationalized in terms of the formation of an interfacial rigid polymer fraction (immobile in DSC and with retarded mobility in BDS) exhibiting higher density. The bulky PLA positioned away from the Ag NPs should exhibit lower density and, consequently, increased free volume. The increased free volume is responsible for fewer chain–chain entanglements (decreased cooperativity) and, therefore, easier diffusion of chains. Such a scenario fits here as well; moreover, our observations are compatible with the above-mentioned increased crystallizability of the nanocomposites compared to unfilled PLA.

### 2.3. Mechanical Evaluation via Tensile and Three-Point Bending Tests

#### 2.3.1. Tensile Properties

**Figure 12:**
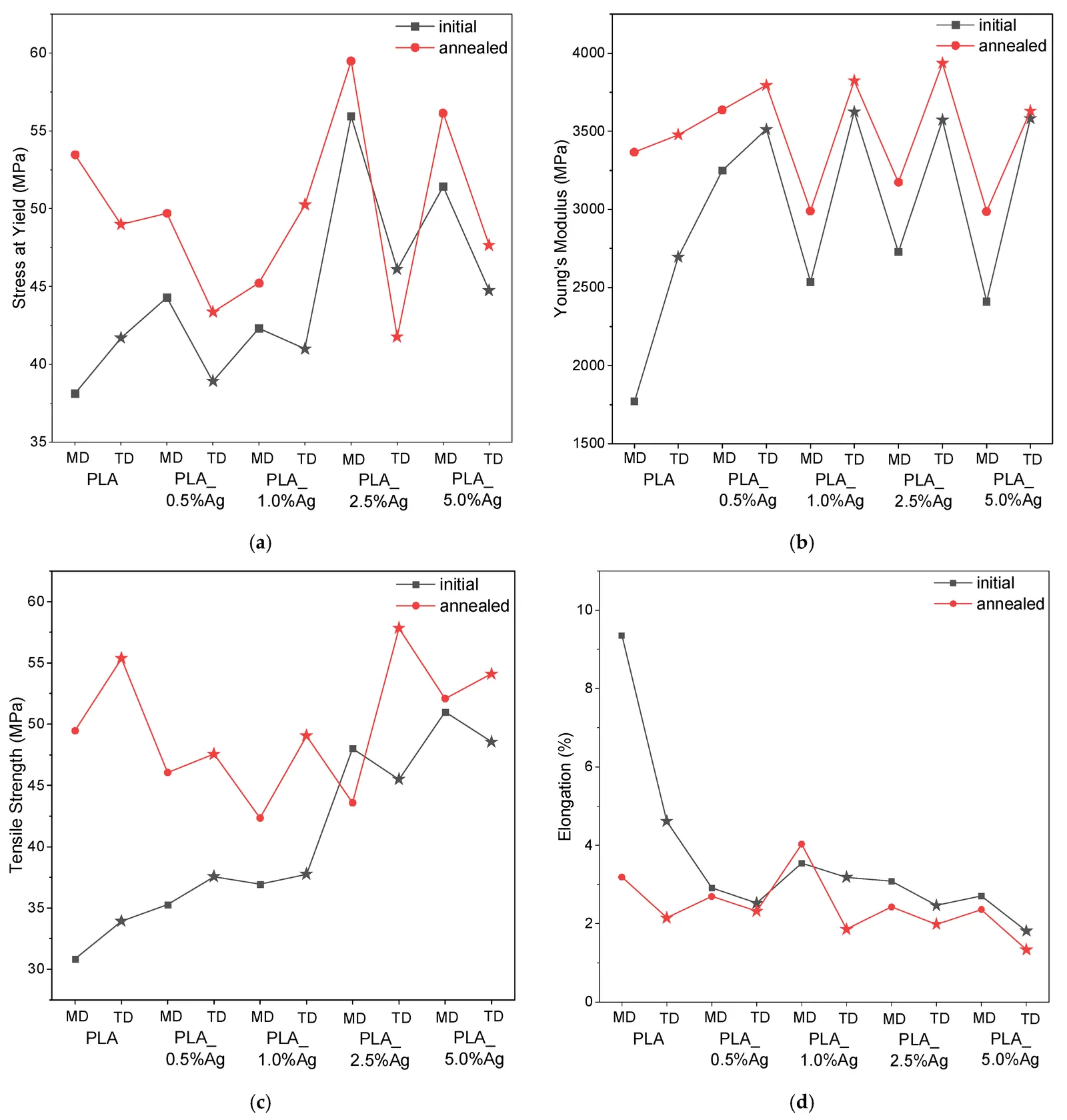
Comparative tensile properties of the PLA cast film sample and PLA nanocomposites before and after annealing: (**a**) stress at yield, (**b**) Young’s modulus, (**c**) stress at break, and (**d**) elongation as a function of the samples. The star shape symbol refers to the transverse direction (TD) and is used as guidance for the eye.

The mechanical performance of cast film samples was thoroughly investigated through stress–strain and three-point bending measurements for both the machine direction (MD) and the transverse direction (TD) (Table S2, Figure S3 and Figure 12). During cast film extrusion, the flow of the melt led to partial orientation of the polymer chains mainly in the MD direction, resulting in the formation of a directionally dependent microstructure. Consequently, the mechanical response of the films significantly differed between MD and TD, leading to increased stiffness and a different stress distribution depending on the loading direction [31,71].

The yield stress (Figure 12a) elevated in the machine direction (MD) compared to the transverse direction (TD), indicating anisotropy due to the orientation of polymer chains during the cast extrusion process. Furthermore, the incorporation of Ag NPs led to an enhancement in the yield stress, particularly for materials containing 2.5 wt.% Ag. Similar behavior has been reported in the literature for extruded PLA-based films by Aversa et al., who demonstrated that the drawing effects during cast extrusion significantly influenced the directional mechanical performance of PLA/poly(butylene adipate-co-terephthalate) (PBAT)/thermoplastic starch (TPS) ternary blends [87]. The elastic modulus (Figure 12b) was higher in the TD compared to the MD. The addition of Ag significantly improved the Young’s modulus of the materials, which can be attributed to the reinforcing effect of the NPs, restricting the elastic deformation of the polymer matrix. Although the literature reports higher elastic modulus values in the MD, especially in polymeric systems due to chain orientation during extrusion [87], the present study reveals an opposite trend (TD > MD). The tensile strength (Figure 12c) showed minor differences between MD and TD, suggesting a relatively uniform distribution of mechanical strength in both directions. The addition of Ag resulted in a noticeable increase in tensile strength, particularly at higher loadings (2.5 and 5 wt.%), and was likely due to the sufficient dispersion of the NPs and the improved interfacial adhesion between the reinforcing phase and the polymer matrix. Higher Young’s modulus and tensile strength values were recorded in the transverse direction (TD), while higher stress at yield and elongation was observed in the machine direction (MD). During cast film extrusion, the polymer chains were preferentially oriented along the MD; however, this orientation may not have been sufficiently strong or uniform to produce the conventional increase in MD modulus and tensile strength. Instead, differences in crystal orientation, lamellar organization and Ag NP dispersion between the two directions may have contributed to the observed behavior. In particular, the crystalline structure may have developed with a different lamellar arrangement in the TD, producing more efficient load transfer and greater resistance to elastic deformation and tensile failure in this direction. The Ag NPs may have reinforced the TD response through interfacial interactions and stress transfer, while any Ag NP-rich regions or aggregates could have locally restricted chain mobility and increased the stiffness.

This restriction and variation in chain mobility was further supported by dielectric spectroscopy analysis. Specifically, the dielectric T_g_ temperature, which monitors the α-relaxation of the dipolar PLA chains, dropped by nearly 5 °C upon addition of up to 2.5 wt.% Ag NPs, before slightly recovering at 5.0 wt.%, which means that the available free volume in the amorphous phase had increased. However, taking the case of elongation at break, despite the decrease in dielectric T_g_, the elongation decreased with Ag NP incorporation, indicating that enhanced segmental mobility alone cannot explain the mechanical response. The simultaneous increase in crystallinity and the possible presence of NPs and induced stress concentration sites may have counteracted the mobility-related effects, restricted macroscopic deformation and contributed to the observed reduction in ductility. Conversely, at the highest concentration (5.0 wt.%), the dielectric T_g_ was slightly increased. Therefore, the overall dielectric response was dictated by a competitive balance between enhanced chain mobility within the less densely packed amorphous zones and structural constraints near the NP surfaces [88,89]. Thus, the slight increase in dielectric T_g_ at 5.0 wt.% Ag suggested greater restriction of segmental motion, which may be consistent with the increased resistance to elastic deformation and the higher stiffness observed at the highest NP loading.

The observed mechanical response should also be considered in relation to the changes in molecular weight and crystallinity induced by increasing Ag NP content. The reduction in molecular weight at higher Ag NP loadings may have reduced the degree of chain entanglement (Figure 11b) and facilitated chain rearrangement and slippage under tensile loading, which could contribute to the observed reduction in elongation at break. At the same time, the increase in crystallinity may have provided additional physical reinforcement by increasing the fraction of rigid crystalline regions and restricting deformation of the amorphous phase, thereby contributing to the higher Young’s modulus and yield stress. The increased crystalline fraction may also have contributed to the tensile strength increasing the resistance of the matrix to deformation. However, the reduction in molecular weight may have partially counteracted this reinforcing effect by weakening the entanglement network. In addition, the direct reinforcing effect of the Ag NPs and their interactions with the PLA matrix may have further increased the stiffness and strength of the films, while the presence of nanoparticle-rich regions or local stress concentrations may have contributed to the reduction in ductility. Thus, the changes observed in the tensile properties with increasing Ag NP content was possibly likely governed by the combined and competing effects of molecular weight, crystallinity, chain mobility, and nanoparticle reinforcement rather than by a single structural parameter. In contrast, the preferentially oriented amorphous chains in the MD may have undergone greater conformational rearrangement during deformation, contributing to the higher elongation observed in this direction. The absence of clearly distinguishable preferential chain directions in the SEM observations (Figure 4) may indirectly support the possibility that the observed mechanical anisotropy cannot be attributed solely to molecular orientation [71,89,90].

The elongation at break (Figure 12d) was higher in the MD compared to the TD, which was caused by the greater ductility along with the processing direction, and this is in agreement with literature reports for cast-extruded polymeric films [87]. However, the incorporation of Ag NPs led to a significant reduction in elongation, as the NPs likely acted as stress concentration points, hindered the mobility of the polymer chains, in which case the crystallinity had also increased (Figure 5d), and appeared as discontinuity parts in the polymer matrix. Overall, the addition of Ag NPs improved the strength and stiffness of PLA cast films while reducing their ductility. At the same time, a pronounced anisotropy between MD and TD was also observed.

Following the annealing experiments (Table S3, Figure S4), a similar overall trend to that in the previous analysis was observed. The yield stress remained higher in the MD compared to the TD. The elastic modulus was higher in the TD than in the MD, and it became even higher at greater loadings. In contrast to the non-annealed samples, the tensile strength was elevated in the TD compared to in the MD, where elongation followed the opposite trend, which indicated that ductility was favored along the processing direction [91,92].

#### 2.3.2. Flexural Properties

**Figure 13:**
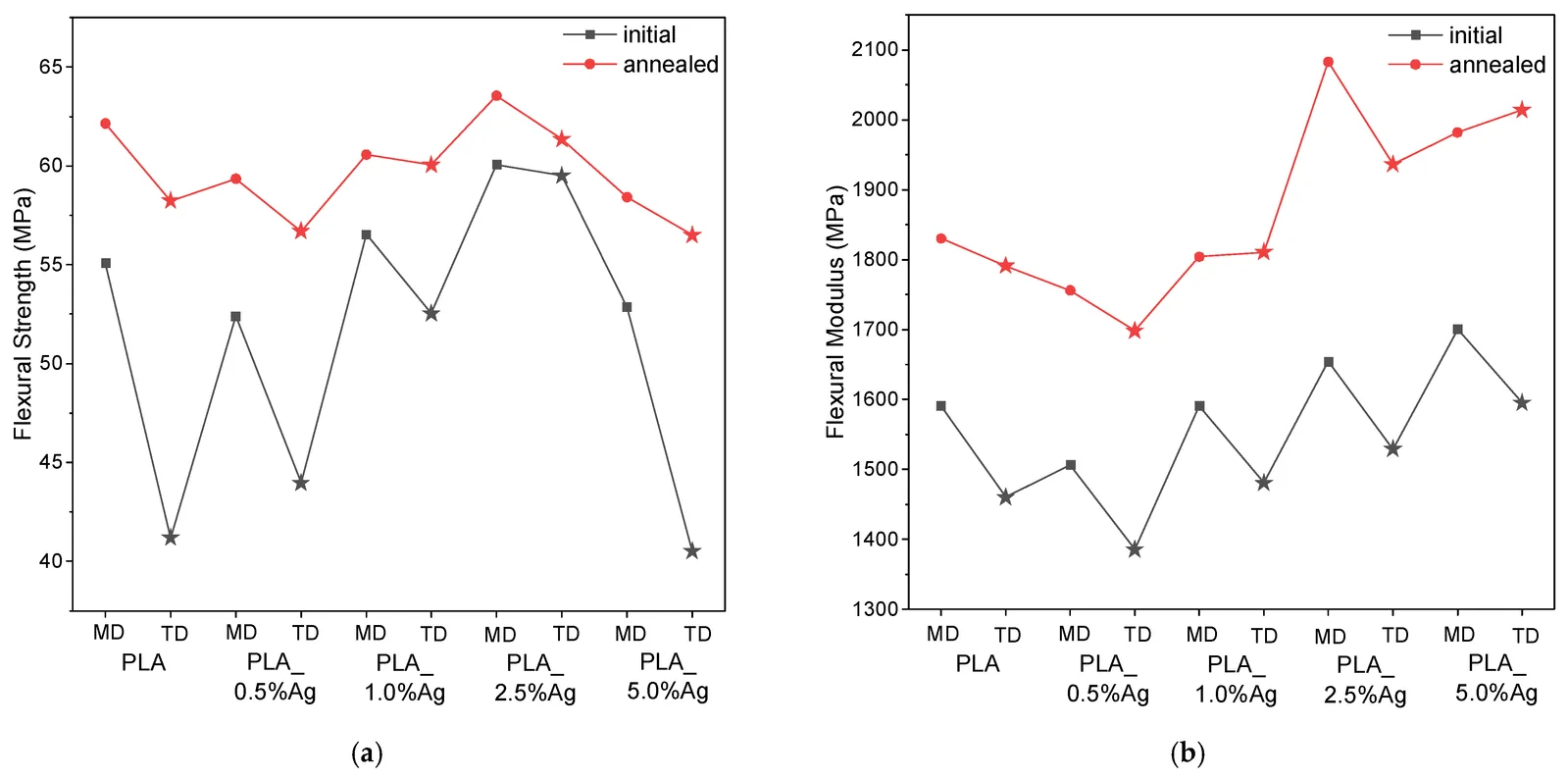
Comparative flexural properties of the PLA cast film sample and PLA nanocomposites before and after annealing: (**a**) flexural strength and (**b**) flexural modulus as a function of the samples. The star shape symbol refers to the transverse direction (TD) and is used as guidance for the eye.

Moving on to the flexural properties, the results (Table S4, Figure S5 and Figure 13) revealed a pronounced anisotropic variation, together with the reinforcing effect of Ag NPs on the bending performance of the PLA films. The flexural strength (Figure 13a) exhibited a significant difference between the MD and TD, with consistently higher values in the MD. This behavior further highlighted the role of processing-induced molecular orientation, which is known to significantly affect the mechanical response of PLA films, particularly under tensile loading, and was expected to influence the flexural behavior [93]. The flexural strength gradually decreased for the low loadings, but for the 2.5 wt.% sample, the highest values were recorded (60.1 ± 5.6 MPa for the MD and 59.5 ± 8.4 Mpa for the TD). This improvement can be attributed to enhanced stress transfer and relatively good NP dispersion within the PLA matrix. At 5.0 wt.% Ag, a slight decrease was observed, particularly in the TD (40.5 ± 6.6), which may have been caused by potential agglomerations [94].

The flexural modulus (Figure 13b) followed a different trend compared to the tensile modulus, showing consistently higher values in the MD than in the TD for all compositions. This suggests that the processing-induced molecular orientation contributed more effectively to flexural resistance than to uniaxial tensile strength. Furthermore, the flexural modulus was increased with the addition of NPs relative to neat PLA, reflecting the reinforcing effect of the rigid NPs and their ability to restrict polymer chain mobility during deformation. This behavior is consistent with previous findings reported by Uwa Orji Uyor et al., who demonstrated that the incorporation of rigid nanofillers (carbon nanotubes decorated with barium titanate) led to an increase in the elastic response of polymer nanocomposites [95]. The highest modulus values for the reported samples were observed at 5.0 wt.% Ag (1700.7 ± 129.4 Mpa for the MD and 1595.6 ± 145.0 Mpa for the TD), indicating that bending stiffness improved with filler loading, even when flexural strength did not follow the same trend.

Following the annealing experiments (Table S5, Figure S6), an overall improvement in both flexural strength and modulus was observed, indicating enhanced structural re-organization of the PLA matrix (Figure 7). The improvement was more pronounced in the MD, suggesting that annealing further reinforced the existing molecular orientation (e.g., neat PLA strength of 62.1 ± 5.6 Mpa in MD vs. 58.2 ± 2.2 Mpa in TD). These results confirmed that annealing promoted the chain rearrangement and increased crystallinity, which further enhanced the flexural resistance and stiffness of the samples.

### 2.4. Surface Properties

**Figure 14:**
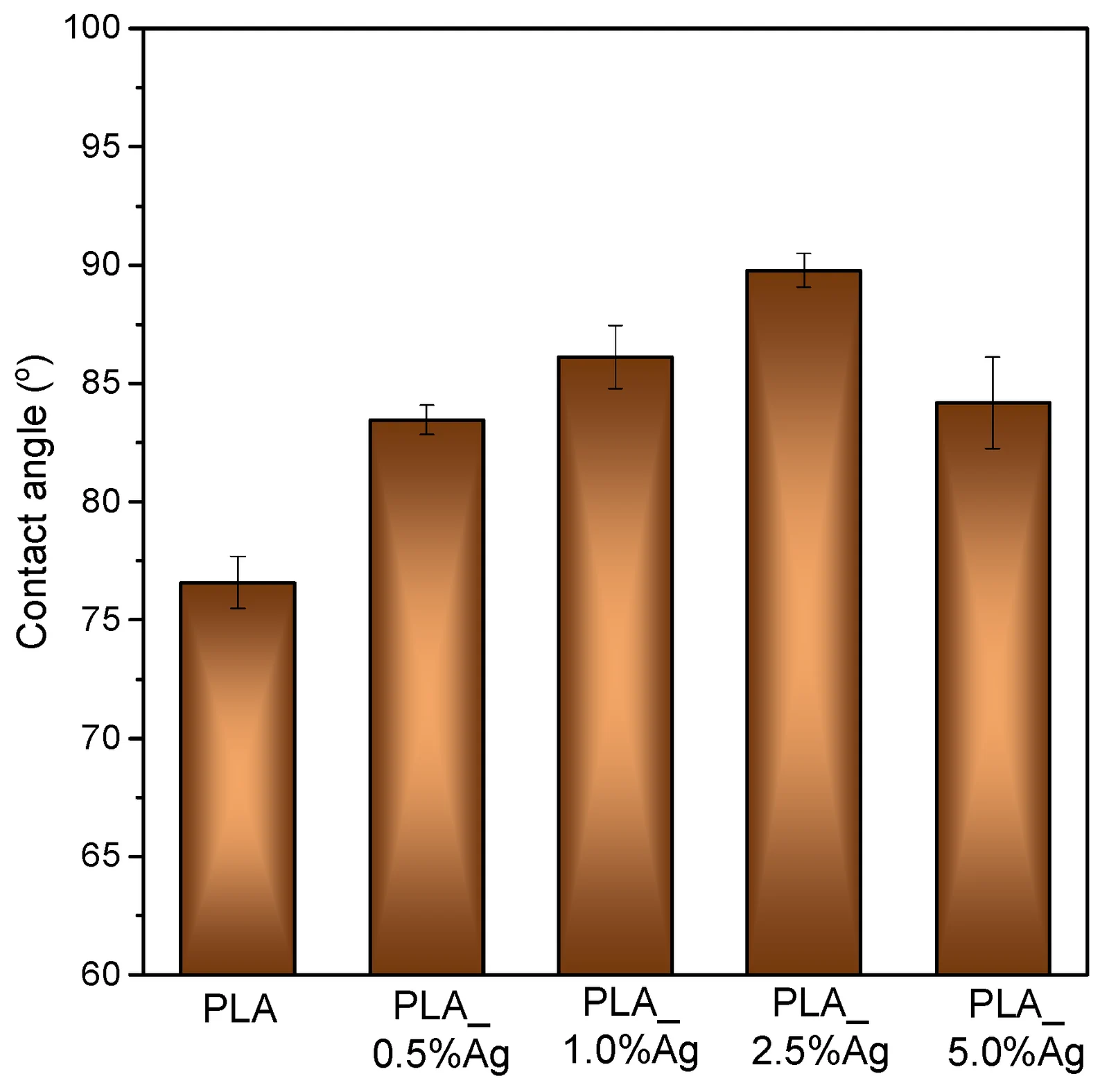
Water contact angle of PLA cast film sample and its nanocomposites.

Water contact angle measurements were employed to evaluate the wettability of PLA cast films and the composite materials (Figure 14). In polymer systems, the measured contact angle is governed by multiple factors, including surface morphology, preparation conditions, chemical structure, roughness and temperature. Neat PLA exhibited a water contact angle of approximately 77°, which is consistent with values reported in the literature [96], confirming the relatively hydrophobic nature of the neat polymer. The incorporation of Ag NPs led to a noticeable increase in the contact angle, indicating enhanced hydrophobicity. Specifically, the addition of 0.5 wt% Ag resulted in a contact angle of 83.5°, while further increasing the Ag content to 1.0 and 2.5 wt% elevated the contact angle to 86° and 90°, respectively. A similar trend in water contact angle was reported by Sztorch et al. for PLA modified with organosilicon spherosilicate derivatives containing alkyl and fluoroalkyl groups. The incorporation of these modifiers increased the hydrophobicity of the PLA surface, which was attributed to the low surface energy of the fluoroalkyl groups and their reduced interactions with water [97]. However, at higher Ag loading (5.0 wt%), a reduction in the contact angle to 84° was observed, suggesting a change in surface characteristics, possibly due to Ag NPs altering the morphology (Figure 4).

## 3. Materials and Methods

### 3.1. Materials

PLA L175, ≥99% (L-isomer), with a melt flow index (MFI) of 8 g/10 min (Flow, 210 °C/2.16 kg) and 3 g/10 min (Flow, 190 °C/2.16 kg), was purchased from Corbion N.V., Gorinchem, The Netherlands. Ag nanopowder and APS 20–40 nm (99.9%) nanoparticles were supplied by ThermoFisher Scientific, Erlenbachweg 2, 76870 Kandel, Germany. All other materials and solvents used were of analytical grade.

### 3.2. Masterbatch Preparation via Solvent Casting

Prior to cast film extrusion, PLA masterbatches containing Ag NPs (0.5, 1.0, 2.5 and 5.0 wt%) were prepared using the solvent casting method. The PLA was first dissolved in chloroform at a concentration of 40 mg/mL, while Ag NPs were dispersed separately in chloroform at 1 mg/mL. Both dispersions were subjected to ultrasonication for 1 h. The two solutions were then combined and sonicated for an additional hour to achieve a uniform NP distribution. The final mixture was placed under vacuum to remove the solvent, resulting in thin nanocomposite films. These films were subsequently cut into small pieces and used as masterbatches during the cast film process.

### 3.3. Preparation of PLA–Silver Nanocomposites via Cast Film Extrusion

**Figure 15:**
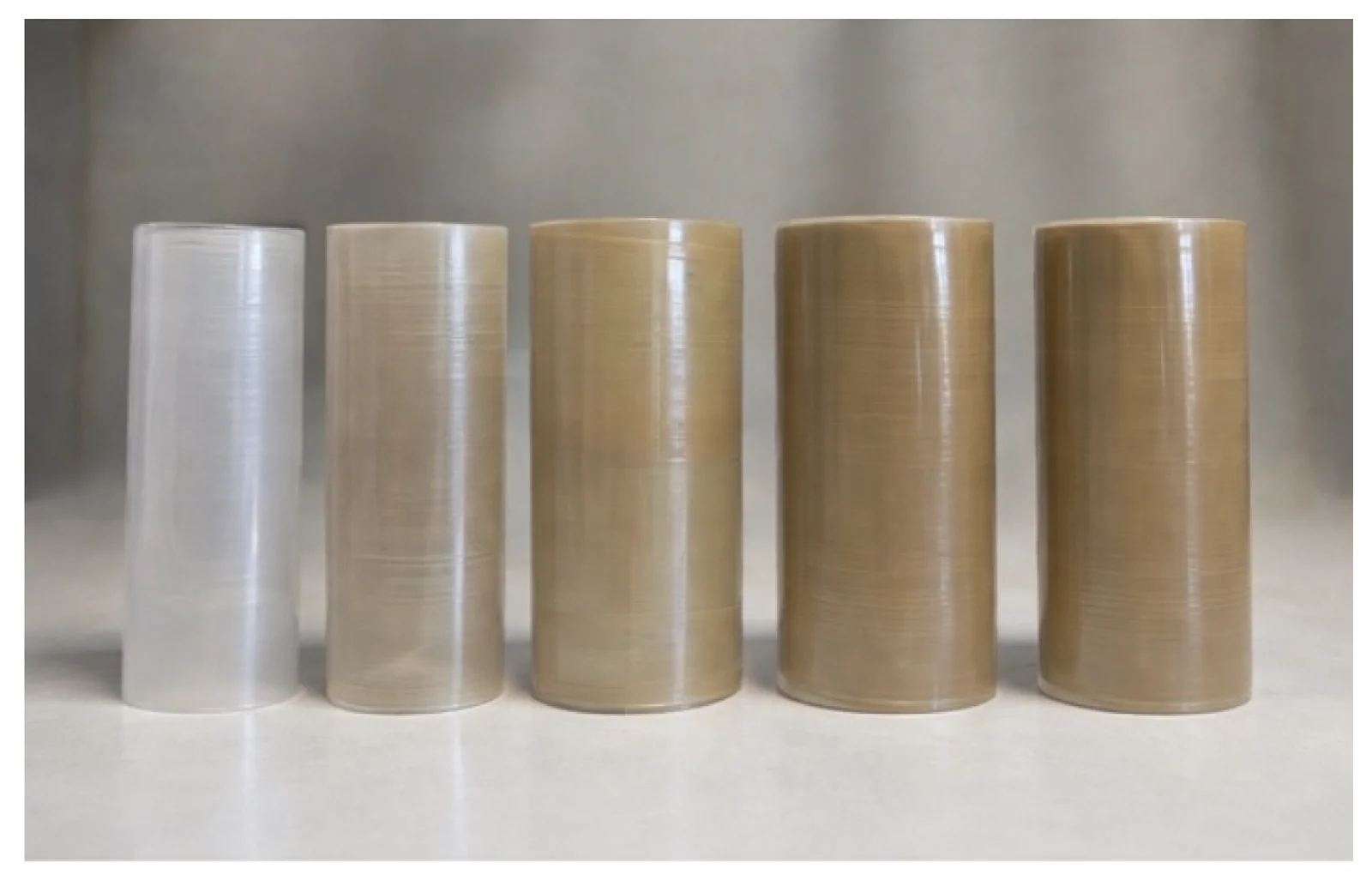
PLA and PLA nanocomposite samples prepared by cast film extrusion. From left to right: neat PLA, 0.5, 1.0, 2.5, and 5.0 wt% Ag NP PLA nanocomposites.

PLA sheets were prepared (Figure 15) using a single-screw cast film extrusion line (Microex Cast, 194 EUR.EX.MA Lab & Pilot Technology, Syncro group, Via Salvator Allende, 7-21049 Tra-195 date (VA)—Italy). Prior to melt mixing, the samples were dried overnight under vacuum at 60 °C. The processing temperature for PLA was set at 190 °C for zones “cylinder 2” and “box”, but “lip” and “cylinder 1” were set at 180 °C. The extrusion speed was set at 60 rpm, while the first and second cylinder were set to 0.2 and 0.4 m/min.

### 3.4. Characterization Techniques

#### 3.4.1. Gel Permeation Chromatography (GPC)

The molecular weight of the materials was determined using GPC/SEC analysis. Measurements were carried out using an Agilent 1260 Infinity II LC system (Agilent Technologies, Santa Clara, CA, USA) equipped with an isocratic G7110B pump, a G7129A automatic vial sampler, and a G7162A refractive index detector (RID). The chromatographic setup included a PLgel 5 μm (50 × 7.5 mm) guard column and two PLgel 5 μm (300 × 7.5 mm) MIXED-C columns. Calibration of the system was performed using poly(methyl methacrylate) (PMMA) standards covering a molecular weight range from 0.535 to 1.591 kg/mol. Samples were dissolved in chloroform, CHCl_3_, at a concentration of 3 mg/mL and filtered through a 0.45 μm PTFE membrane filter to remove any insoluble particles. An injection volume of 20 μL was used for each run, with a total elution time of 30 min. The temperatures of both the column compartment and the RID detector were maintained at 40 °C during the analysis.

#### 3.4.2. Intrinsic Viscosity

Intrinsic viscosity [η] measurements were conducted using an Ubbelohde viscometer (capillary 0c) at 25 °C, with chloroform as the solvent. The solutions were filtered through a disposable Teflon membrane to remove any solid residues. Intrinsic viscosity was calculated by applying the Solomon–Cuita equation:

(2) $$\left[\eta \right]=\frac{{\left(2\left\{\frac{t}{t_{o}}-\ln\left(\frac{t}{t_{o}}\right)-1\right\}\right)}^{1/2}}{c}$$

where *c* is the solution concentration, *t* is the flow time of the solution and *t_o_* is the flow time of the pure solvent. Each measurement was performed in triplicate.

#### 3.4.3. Nuclear Magnetic Resonance (NMR)

The NMR spectra were conducted in deuterated chloroform for the structural study of PLA cast film samples. An Agilent 500 spectrometer was used (Agilent Technologies, Santa Clara, CA, USA) at room temperature. Spectra were calibrated using the peaks of the residual solvents.

#### 3.4.4. Attenuated Total Reflectance Fourier-Transform Infrared Spectroscopy (ATR-FTIR)

FTIR spectra were recorded using an IRTracer-100 spectrophotometer (Shimadzu, Kyoto, Japan), equipped with a QATR™ 10 single-reflection ATR accessory. Spectra were acquired over the 4000–500 cm^−1^ wavenumber region, with a resolution of 2 cm^−1^, and a total of 32 co-added scans. Prior to analysis, all spectra were normalized to ensure consistent comparison.

#### 3.4.5. Scanning Electron Microscopy (SEM)

Scanning electron microscopy (SEM) was employed to examine the interfacial (cross-section) section of the PLA cast films and PLA/Ag NPs. SEM analyses were performed using a field-emission scanning electron microscope (JEOL, Tokyo, Japan, JSM 7610F) operating at an accelerating voltage of 5 kV. Prior to observation, all specimens were coated with a thin carbon layer to enhance electrical conductivity.

#### 3.4.6. Differential Scanning Calorimetry (DSC)

Differential scanning calorimetry (DSC) analysis was performed using a PerkinElmer Pyris Diamond DSC (Solingen, Germany), which had been calibrated with pure indium, tin and zinc standards. The instrument was equipped with a PerkinElmer Intracooler 2 cooling accessory (Solingen, Germany) and operated under a nitrogen atmosphere. Samples of 5.0 ± 0.5 mg were sealed in aluminum pans prior to testing to examine the thermal transitions of the polymeric materials. To eliminate the thermal history of the materials, each sample underwent an initial heating cycle up to T_m_ + 30 °C and held for 3 min. Following this step, the materials were quenched at 150 °C/min to 10 °C and subsequently reheated at 20 °C/min to determine the glass transition temperature (T_g_), cold crystallization temperature (T_cc_), and melting temperature (T_m_). A final cooling step was performed from 200 °C to 0 °C at 10 °C/min to observe the melt crystallization temperature (T_c_).

Isothermal melt crystallization tests were also conducted to assess preliminary crystallization kinetics. The samples were first heated to a temperature of 30 °C above their T_m_ and held for 3 min to remove any residual thermal history, following a cooling step to 120 °C at the maximum cooling rate achievable by the instrument. A final heating step to (T_m_ + 30 °C) was applied at 20 °C/min.

#### 3.4.7. X-Ray Diffraction (XRD)

Samples were subjected to X-ray diffraction measurements with the MiniFlex II XRD system from Rigaku Co. (Tokyo, Japan) with CuKa radiation (λ = 0.154 nm) in the angle 2θ range from 5 to 45° at a scanning rate of 1°/min. The sample holder for the measurements was approximately 32 mm in diameter.

#### 3.4.8. Polarized Light Microscopy (PLM)

The semicrystalline morphology was also studied during isothermal melt crystallization optically, employing polarized light microscopy (PLM). The PLM images were taken during isothermal crystallization from the melt state by means of a Nikon Optiphot-1 polarizing microscope (Nikon, Tokyo, Japan) equipped with a Linkam THMS 600 heated stage (Linkam Scientific Instruments Ltd., Redhill, UK), a Linkam TP91 control unit, and a Jenoptic Gryphax Naos camera (Jenoptik, Jena, Germany), accompanied by Jenoptic Gryphax software, version 2.1.

#### 3.4.9. Broadband Dielectric Spectroscopy (BDS)

BDS was employed to investigate, among others, the molecular dynamics of the samples. The measurements were performed using a Novocontrol BDS setup consisting of an Alpha frequency response analyzer coupled with a Novocontrol Quatro liquid nitrogen cryosystem (Novocontrol GmbH, Montabaur, Germany) under a nitrogen atmosphere. The measurements were conducted on samples placed between finally polished brash electrodes (disks) employing thin silica spacers. The samples were melt-quenched in the sandwich-like capacitor form, containing initially amorphous polymers. The complex dielectric permittivity ε*(f,T) and the conductivity in the alternate current, σ_AC_, were recorded isothermally at various temperatures, T, ranging from −120 to 120 °C upon heating at steps of 5 or 10 K and over a broad frequency, f, range from 10^−1^ to 10^6^ Hz.

#### 3.4.10. Tensile Properties

The tensile properties of the cast film samples were evaluated using a Shimadzu EZ Test Tensile Tester (Model EZ-LX) (1, Nishinokyo Kuwabara-cho, Nakagyo-ku, Kyoto 604-8511, Japan) equipped with a 2 kN load cell, in accordance with ASTM D882, at a crosshead speed of 5 mm/min. Tensile specimens were prepared from the cast films and cut along both the machine direction (MD) and the transverse direction (TD) in order to assess the potential anisotropy induced during the film casting process. Dumbbell-shaped specimens (central portion: 5 mm wide × 0.5 mm thick, 22 mm gauge length) were prepared by cutting the films using a Wallace cutting press. At least five measurements were performed for each sample and testing direction, and mean values were calculated for the tensile parameters. The tensile properties were also examined for the annealed samples (at 110 °C for 1 h).

#### 3.4.11. Flexural Properties

Three-point bending tests were performed using a Shimadzu EZ Flexural Tester (Model EZ-LX) equipped with a 2 kN load cell, in accordance with ASTM D790-17. Flexural specimens were prepared from the cast films and cut either parallel or perpendicular to the film casting direction, corresponding to the machine direction (MD) and the transverse direction (TD), respectively, in order to investigate possible processing-induced anisotropy. The specimens had dimensions of 12.7 mm in width and 1.6 mm in thickness and were tested flatwise on the support span, with a support span-to-depth ratio of 16:1 (±1 tolerance). For each sample type and testing direction, at least five measurements were carried out, and the reported values of flexural modulus and flexural strength represented the mean of the obtained results. The flexural properties were also examined for the annealed samples at 110 °C for 1 h.

#### 3.4.12. Water Contact Angle

The water contact angle was determined using the sessile drop technique with an Ossila contact angle goniometer (Model L2004A1; Ossila Ltd., Sheffield, UK). All measurements were performed in triplicate, and the reported values correspond to the average contact angle, along with the associated standard deviation (SD).

## 4. Conclusions

PLA/Ag nanocomposites were successfully developed using the cast film extrusion technique. The results confirmed that PLA was highly susceptible to thermomechanical degradation during processing, as evidenced by the reduction in both the molecular weight and intrinsic viscosity, while Ag NPs further restricted the chain scission reactions, suggesting a possible contribution to thermomechanical stability through cast film extrusion. ^1^H NMR and FTIR analysis confirmed the structural PLA backbone without any chemical modification. Extrusion induced chain orientation for the neat PLA, while Ag NP incorporation modified the interfacial cross section morphology, confirming effective dispersion on the PLA matrix. Thermal analysis showed that Ag NPs did not significantly affect T_g_ or T_m_ but acted as nucleating agents, increasing crystallinity. XRD confirmed the successful incorporation of Ag NPs into the polymer matrix, without causing any changes to the crystalline structure of PLA. Annealing experiments further promoted crystallization without inducing new phases. Isothermal melt crystallization experiments and PLM studies confirmed the nucleation ability of the Ag NPs, where the crystallization half-time was significantly reduced for the nanocomposites sheets, implying improved crystallization rates of the isothermal melt crystallization kinetics. The molecular dynamics findings obtained by BDS support that the nucleation effect was indirect. In particular, from the full dielectric relaxation mapping, it was revealed that the presence of Ag NPs led to accelerated segmental dynamics (decreasing dielectric T_g_) and to decreased fragility. The latter is compatible with the decreased chain–chain cooperativity/entanglement level and, possibly, the increased free polymer volume. Combining these effects with the findings on crystallinity, we can conclude that the favored crystallizability of the nanocomposites originates from both the nucleating role of Ag and the enhanced diffusion of chains due to the increased free volume. Regarding the mechanical properties, Ag NPs improved the tensile strength and stiffness of PLA, while reducing ductility, with the anisotropy between the MD and TD being governed by processing-induced orientation during the cast film extrusion process. Annealing further enhanced the stiffness through increased crystallinity, where flexural tests followed similar trends. Last but not least, the presence of Ag NPs influenced the wettability of PLA films by increasing the hydrophobicity of the samples at intermediate compositions. Overall, the results demonstrate that the cast film PLA/Ag nanocomposite sheets exhibit improved structural, thermal, mechanical, and surface properties, making them promising materials for advanced industrial applications.

## Figures and Tables

**Table 1:** Thermal transitions of PLA cast film sample and its nanocomposites.

Sample	As Received		Quenched					Cooling		
	ΔH_m_ (J/g)	T_m_ (°C)	T_g_ (°C)	ΔH_cc_ (J/g)	T_cc_ (°C)	ΔH_m_ (J/g)	T_m_ (°C)	ΔH_c_ (J/g)	CF_c_ (%)	T_c_ (°C)
PLA	31.0	172.5	60.0	26.8	107.7	31.9	173.4	1.1	1.2	105.7
PLA_0.5%Ag	30.1	172.2	58.9	27.8	110.1	28.6	172.5	4.3	4.6	99.2
PLA_1.0%Ag	31.6	171.5	59.0	31.0	107.4	33.2	171.8	8.6	9.3	97.2
PLA_2.5%Ag	33.0	172.2	60.7	31.3	105.7	32.2	172.8	16.7	18.0	97.8
PLA_5.0%Ag	37.7	171.5	59.8	30.4	104.0	33.5	172.2	19.1	20.5	99.5

## Data Availability

The data supporting this article have been included as part of the Supplementary Materials File. The rest of the data supporting this article will be available upon request to the corresponding authors, uniquely in the frame of private communication.

## Supplementary Materials

The following supporting information can be downloaded at https://www.mdpi.com/article/10.3390/molecules31213808/s1. The Supporting Information (SI) file includes additional data and analysis results on GPC, tensile and flexural tests. Table S1: GPC and intrinsic viscosity results; Figure S1: (a) Temperature peaks and (b) crystalline fractions of cold (T_cc_, CF_cc_) and melt (T_c_, CF_c_) crystallization of PLA cast film samples; Figure S2: Isothermal crystallization of the samples at 120 °C. (a) Exothermic crystallization curves, (b) relative degree of crystallinity as a function of time, and (c) subsequent heating traces; Table S2: Tensile data of PLA cast film sample and PLA nanocomposites; Figure S3: Tensile properties of PLA cast film sample and its nanocomposites. (a) Stress at yield, (b) Young’s Modulus, (c) Stress at break, and (d) Elongation; Table S3: Tensile data of PLA cast film sample and PLA nanocomposites after annealing; Figure S4: Tensile properties of PLA cast film sample and PLA nanocomposites after annealing. (a) Stress at yield, (b) Young’s Modulus, (c) Stress at break, and (d) Elongation as a function of the samples; Table S4: Flexural data of PLA cast film sample and PLA nanocomposites; Figure S5: Flexural properties of PLA nanocomposite materials. (a) Flexural strength and (b) Flexural Modulus of the samples. The Flexural Strength indicates the maximum flexural stress the material can withstand during the experiments; Table S5: Flexural data of PLA cast film sample and PLA nanocomposites after annealing; Figure S6: Flexural properties of cast film samples after annealing. (a) Flexural strength, and (b) flexural modulus of samples. The Flexural Strength indicates the maximum flexural stress the material can withstand during the experiments.
