# Supplementary material for: Unlocking Nanocomposites via Cast Film Extrusion: Processing–Structure–Property Relationships of PLA Sheets Containing Silver Nanoparticles
Source: Molecules. 2026 Oct 22;31(21):3808. doi: 10.3390/molecules31213808 (PMC13764800; doi:10.3390/molecules31213808)
Supplement: Supplementary file 1 [file molecules-31-03808-s001.zip › molecules-4623428-supplementary.pdf]

Unlocking Nanocomposites via Cast Film Extrusion: Processing - Structure - Property Relationships of PLA sheets containing silver nanoparticles

Kyriaki Lazaridou [1], Rafail O. Ioannidis [1], Panagiotis A. Klonos [1, 2], Apostolos Kyritsis [2] and Dimitrios N. Bikiaris [1,*]

[1] Laboratory of Polymer Chemistry and Technology, Department of Chemistry, Aristotle University of Thessaloniki, 54124 Thessaloniki, Greece
[2] Department of Physics, National Technical University of Athens, Zografou Campus, 15780 Athens, Greece
* Correspondence: dbic@chem.auth.gr

Supplementary material

Table S1: GPC and intrinsic viscosity results.

Sample	$\overline{M_n}$ (g/mol)	Đ	[η] (dL/g)
**PLA**	109.800	2.03	1.56
**PLA/0.5%Ag**	138.800	1.27	1.57
**PLA/1.0%Ag**	112.100	1.49	1.57
**PLA/2.5%Ag**	100.200	1.74	1.52
**PLA/5.0%Ag**	99.900	1.73	1.37

$\overline{M_n}$: Number Average Molecular Weight, Đ: dispersity

*PLA before extrusion; $\overline{M_n}$ = 140 Kg/mol, Đ = 1.17 and [η] = 1,64 dL/g.

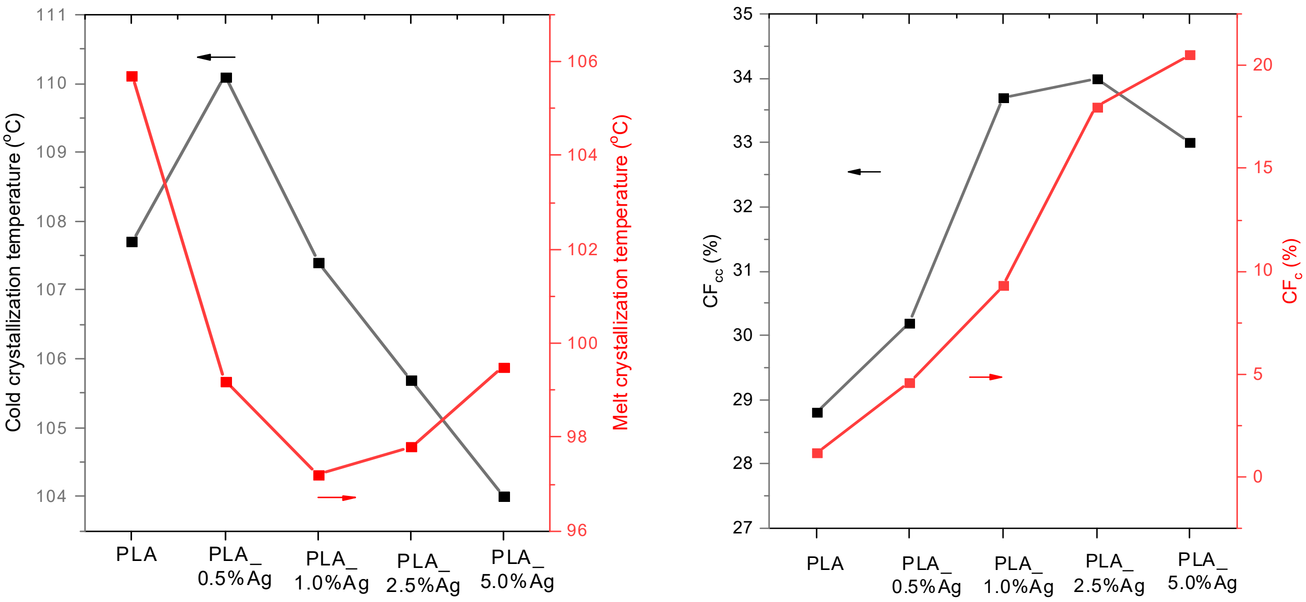

(a) (b)

Figure S1. (a) Temperature peaks and (b) crystalline fractions of cold (T_{cc}, CF_{cc}) and melt (T_c, CF_c) crystallization of PLA cast film samples.

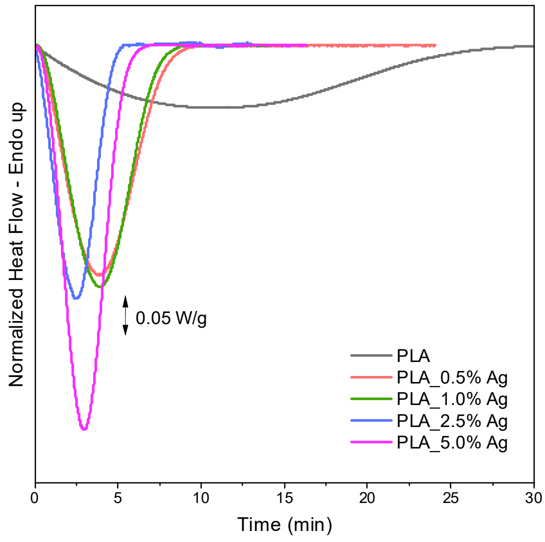

(a)

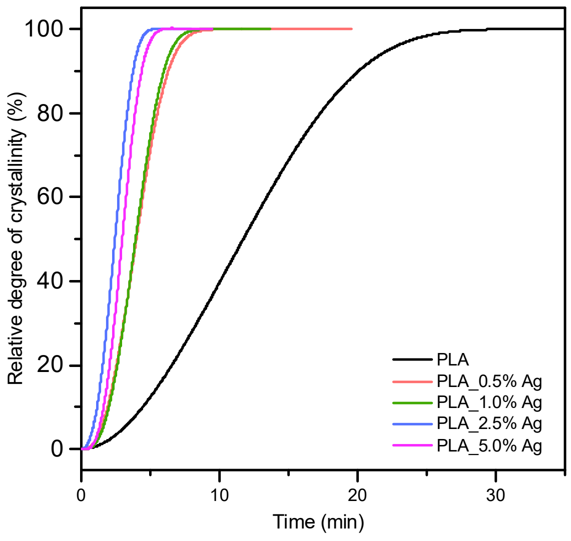

(b)

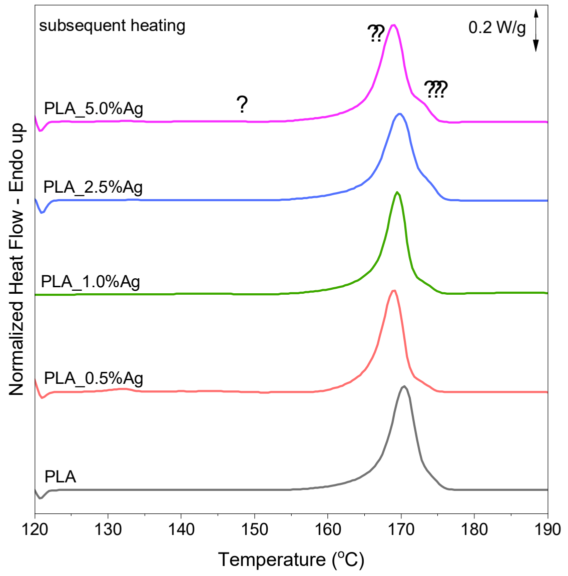

(c)

Figure S2. Isothermal crystallization of the samples at 120 °C. (a) Exothermic crystallization curves, (b) relative degree of crystallinity as a function of time, and (c) subsequent heating traces.

Table S2: Tensile data of PLA cast film sample and PLA nanocomposites.

Sample	Tensile Strength (MPa)		Tensile Stress at Yield (MPa)		Elongation (%)		Young's Modulus (MPa)	
	MD	TD	MD	TD	MD	TD	MD	TD

**PLA**	30.8 ± 7.2	33.9 ± 6.4	38.1 ± 1.3	41.7 ± 4.8	9.6 ± 3.8	4.6 ± 3.0	1771.2 ± 427.0	2694.1 ± 750.7
**PLA/0.5%Ag**	35.3 ± 5.8	37.6 ± 9.2	44.3 ± 5.8	38.9 ± 8.8	2.9 ± 0.3	2.5 ± 0.9	3250.3 ± 415.0	3512.2 ± 934.2
**PLA/1.0%Ag**	36.9 ± 4.3	37.8 ± 5.6	42.3 ± 4.5	40.1 ± 4.5	3.5 ± 3.6	3.2 ± 3.2	2534.3 ± 451.7	3624.9 ± 445.3
**PLA/2.5%Ag**	48.0 ± 4.0	45.5 ± 5.5	55.9 ± 0.8	46.1 ± 7.7	3.1 ± 0.3	2.5 ± 0.8	2727.7 ± 329.8	3571.4 ± 187.5
**PLA/5.0%Ag**	51.0 ± 1.1	48.6 ± 3.3	51.4 ± 5.1	44.7 ± 4.7	2.7 ± 1.8	1.8 ± 0.5	2410.5 ± 536.4	3579.9 ± 445.5

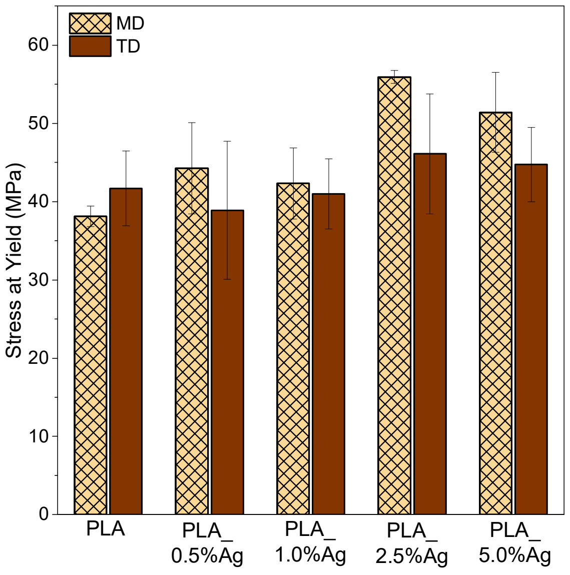

(a)

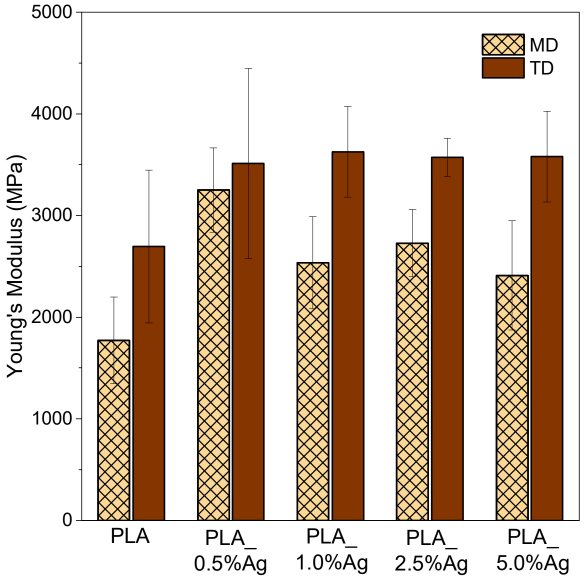

(b)

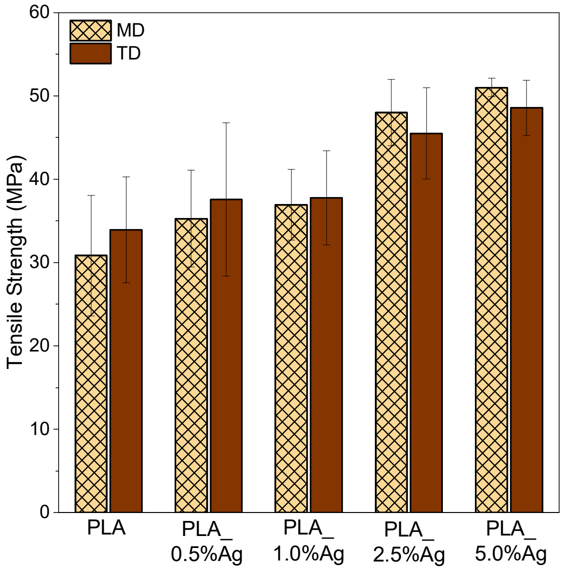

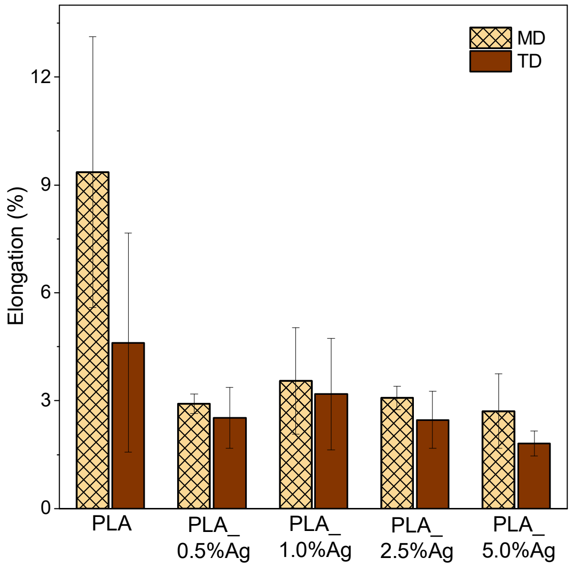

(c) (d)

Figure S3: Tensile properties of PLA cast film sample and its nanocomposites. (a) Stress at Yield, (b) Young's Modulus, (c) Stress at break, and (d) Elongation.

Table S3: Tensile data of PLA cast film sample and PLA nanocomposites after annealing.

Sample	Tensile Strength (MPa)		Tensile Stress at Yield (MPa)		Elongation (%)		Young's Modulus (MPa)	
	MD	TD	MD	TD	MD	TD	MD	TD
**PLA**	49.5 ± 5.1	55.4 ± 4.6	53.5 ± 6.1	49.0 ± 6.9	3.19 ± 0.96	2.15 ± 0.1	3366.4 ± 84.6	3476.8 ± 383.2
**PLA/0.5%Ag**	46.1 ± 7.0	47.6 ± 8.8	49.7 ± 5.4	43.4 ± 6.1	2.70 ± 0.64	2.32 ± 0.27	3636.2 ± 469.9	3794.8 ± 185.9
**PLA/1.0%Ag**	42.3 ± 7.1	49.1 ± 3.7	45.2 ± 7.7	50.2 ± 1.4	4.03 ± 0.53	1.86 ± 0.25	2989.9 ± 449.8	3823.6 ± 347.0
**PLA/2.5%Ag**	43.6 ± 4.4	57.9 ± 3.0	59.5 ± 6.4	41.8 ± 3.3	2.42 ± 0.19	1.98 ± 0.20	3172.6 ± 681.2	3935.0 ± 386.1
**PLA/5.0%Ag**	52.1 ± 2.0	54.1 ± 1.3	56.1 ± 2.3	47.6 ± 4.8	2.32 ± 0.32	1.33 ± 0.10	2986.6 ± 211.0	3629.5 ± 338.1

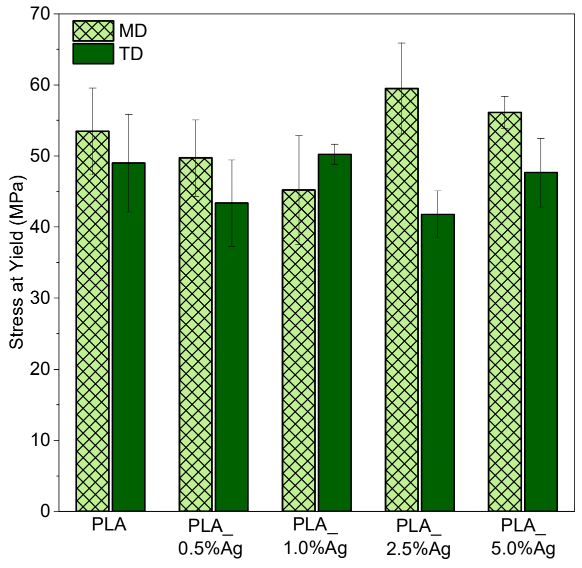

(a)

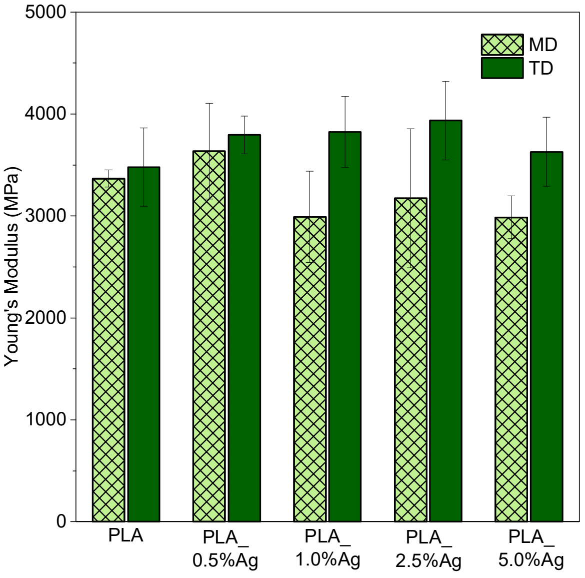

(b)

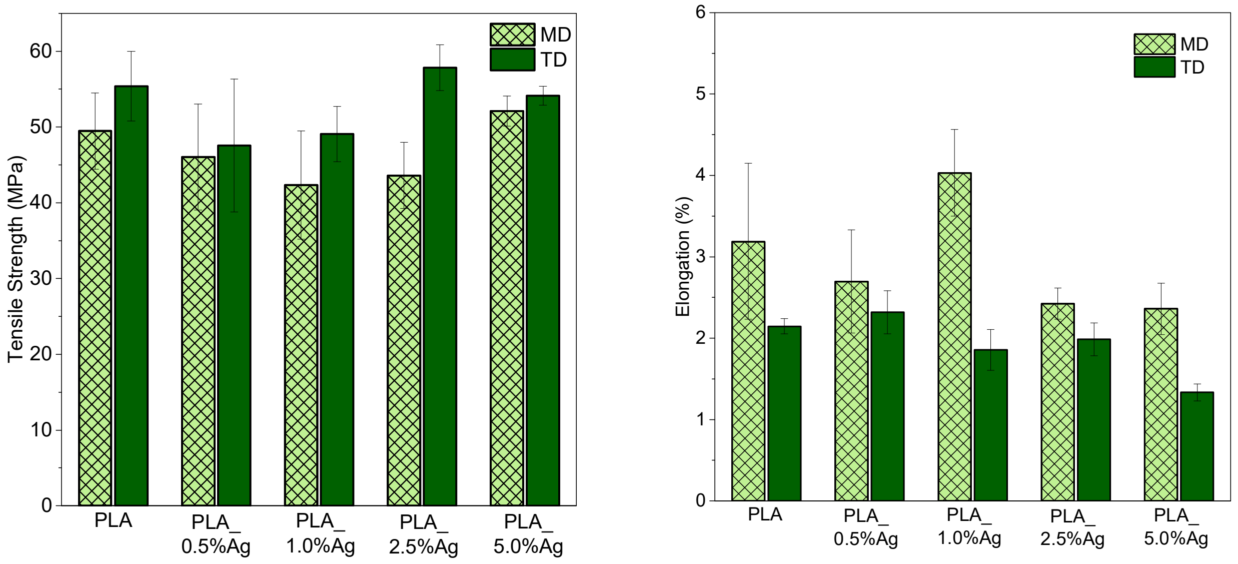

(c) **(d)**

Figure S4: Tensile properties of PLA cast film sample and PLA nanocomposites after annealing. (a) Stress at Yield, (b) Young's Modulus, (c) Stress at break, and (d) Elongation as a function of the samples.

Table S4: Flexural data of PLA cast film sample and PLA nanocomposites.

**Sample**	**Flexural Strength (MPa)**		**Flexural Modulus (MPa)**	
	**MD**	**TD**	**MD**	**TD**
**PLA**	55.1 ± 8.1	41.2 ± 9.6	1590.7 ± 230.0	1460.1 ± 294.4
**PLA_0.5%Ag**	52.4 ± 4.7	44.0 ± 7.0	1506.6 ± 321.7	1385.2 ± 215.6
**PLA_1.0%Ag**	56.5 ± 5.2	52.5 ± 7.0	1591.2 ± 272.0	1480.9 ± 196.4
**PLA_2.5%Ag**	60.1 ± 5.6	59.5 ± 8.4	1654.6 ± 471.0	1529.0 ± 405.6
**PLA_5.0%Ag**	52.9 ± 5.6	40.5 ± 6.6	1700.7 ± 129.4	1595.6 ± 145.0

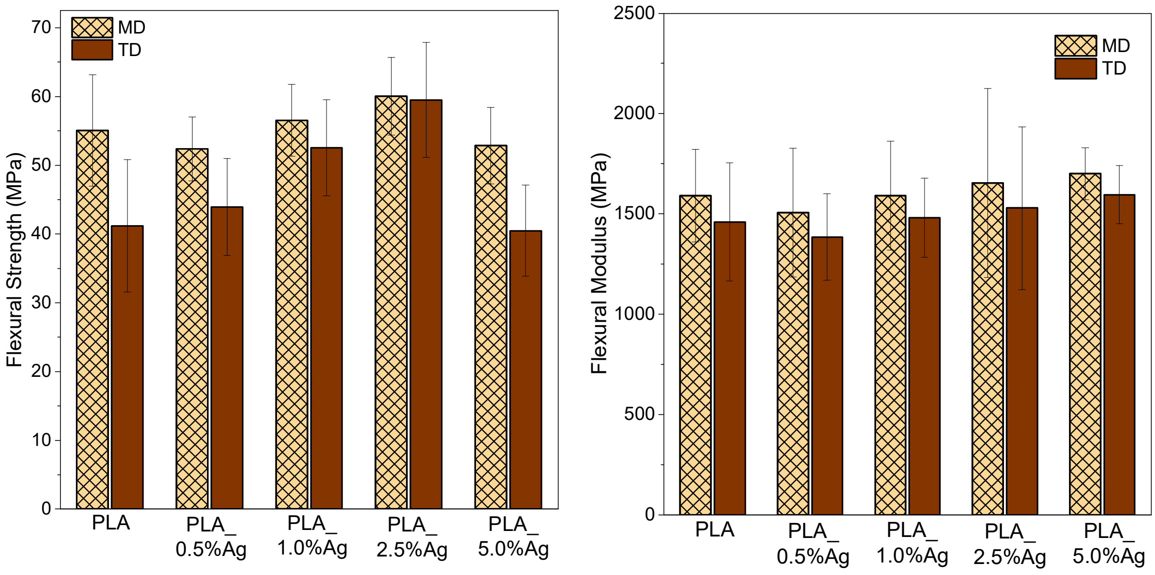

(a) **(b)**

Figure S5: Flexural properties of the PLA nanocomposites materials. (a) Flexural strength, and (b) Flexural Modulus of the samples. The Flexural Strength indicates the maximum flexural stress the material can withstand during the experiments.

Table S5: Flexural data of PLA cast film sample and PLA nanocomposites after annealing.

Sample	Flexural Strength (MPa)		Flexural Modulus (MPa)	
	MD	TD	MD	TD
**PLA**	62.1 ± 5.6	58.2 ± 2.2	1830.3 ± 181.7	1791.0 ± 236.5
**PLA/0.5%Ag**	59.4 ± 7.0	56.7 ± 7.7	1756.1 ± 386.2	1698.5 ± 193.7
**PLA/1.0%Ag**	60.6 ± 3.7	60.1 ± 8.5	1804.0 ± 231.6	1810.5 ± 312.1
**PLA/2.5%Ag**	63.5 ± 3.7	61.4 ± 6.0	2083.1 ± 217.1	1936.7 ± 183.0
**PLA/5.0%Ag**	58.4 ± 4.4	56.5 ± 9.2	1982.0 ± 246.2	2013.8 ± 167.5

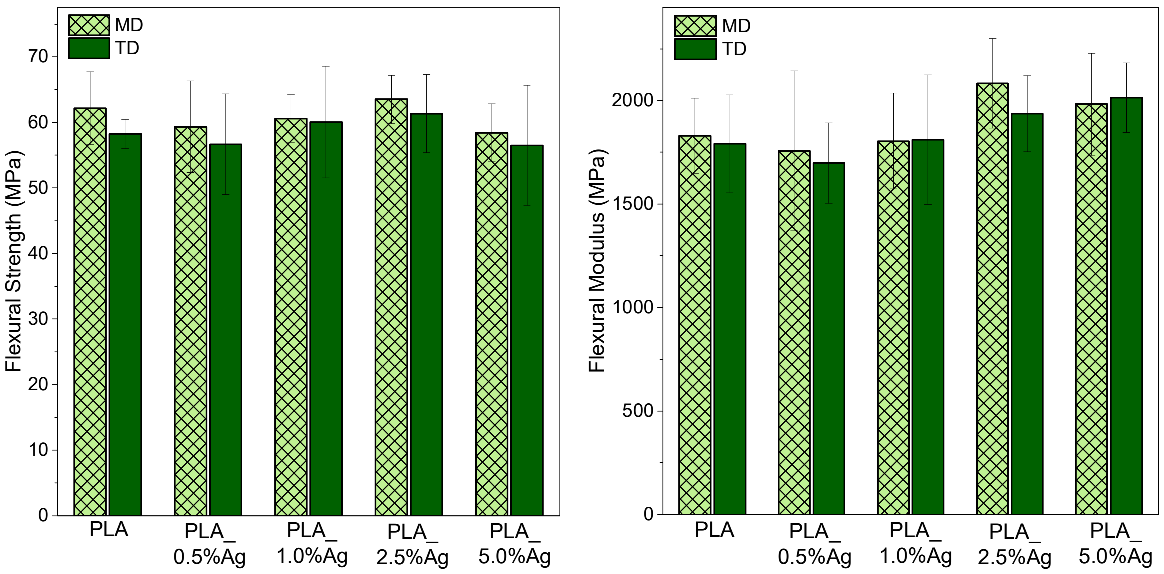

(a) **(b)**

Figure S6: Flexural properties of the cast film samples after annealing. (a) Flexural strength, and (b) Flexural Modulus of the samples. The Flexural Strength indicates the maximum flexural stress the material can withstand during the experiments.
